# Multi-omics profiling of chromatin accessibility and H3K27ac reveals super-enhancer–mediated regulatory networks governing endometrial receptivity in goats

**DOI:** 10.1186/s40104-025-01318-2

**Published:** 2026-01-09

**Authors:** Zhipeng Sun, Junyin Zhao, Yuhao Liao, Yuqin Cheng, Houmo Yu, Mingming Wang, Xingqiang Fang, Songjian Yang, Yongju Zhao

**Affiliations:** College of Animal Science and Technology, Southwest University, Chongqing Key Laboratory of Herbivore Science, Chongqing Engineering Research Center for Herbivore Resource Protection and Utilization, No. 2 Tiansheng Rd., Beibei District, Chongqing, 400715 China

**Keywords:** Chromatin accessibility, Endometrial receptivity, Goat, H3K27ac, Transcriptional regulation

## Abstract

**Background:**

Endometrial receptivity (ERE) is a transient uterine state that determines the success of blastocyst implantation; however, the epigenomic regulation underlying ERE establishment in goats remains unclear. Here, we profiled transcriptional and epigenomic features of endometrial tissues from pregnant goats during the peri-implantation window and nonpregnant control goats in the regressed luteal phase to uncover the transcriptional regulatory networks responsible for ERE establishment in goats, utilizing RNA-seq, ATAC-seq, and H3K27ac CUT&Tag.

**Results:**

A total of 3,143 differentially expressed genes (DEGs) were identified, accompanied by significant alterations in chromatin accessibility and H3K27ac modifications between receptive and non-receptive endometria. The targeted genes associated with these epigenetic changes were significantly enriched in pathways related to cell adhesion, immune tolerance, and embryo attachment. Motif enrichment and transcription factor (TF) footprinting analyses identified members of the FOS/JUN, SOX, HNF1, CEBP, and BATF families as candidate regulators, implicating downstream genes involved in ERE establishment, including *SPP1*, *FOXO1*, *KLF4/6*, *STAT1*, *IFI6*, *ITGB8*, *PLAC8*, *DUSP4*, *NR1D1*, *ISG15*, *RUFY4*, and *PIK3R3*. In addition, numerous super-enhancers were identified, indicating regions of high regulatory activity and potential long-range gene-enhancers interactions in the endometrium. Integration of multi-omics datasets revealed a strong correlation (*r* > 0.7) among chromatin accessibility, H3K27ac activation, and the expression of 172 DEGs. Furthermore, a set of hub genes (*KLF6*, *IFI6*, *MCL1*, *SDC4*, *SUSD6*, *MAFF*, and *IL6R*) that appear to coordinate TF binding and distal super-enhancers activity associated with ERE establishment.

**Conclusions:**

Our data provided an integrated epigenomic atlas of endometrial receptivity establishment in goats and identify candidate regulatory elements and transcription factors that may orchestrate uterine preparation for implantation. These findings offer valuable insights and testable targets for improving fertility in ruminant livestock.

**Supplementary Information:**

The online version contains supplementary material available at 10.1186/s40104-025-01318-2.

## Introduction

Improving female fertility remains a critical economic objective in livestock production [1]. Fertility depends on the ability of the uterus to support blastocyst implantation, placentation, and normal parturition [2]. However, early embryonic loss—mainly due to implantation failure—remains a major limitation in animal reproduction. Successful implantation requires precise coordination between the developmental competence of the blastocysts and the endometrium receptivity (ERE) of the uterus [3–6]. Previous studies have shown that when morphologically normal blastocysts are transferred, implantation failure is usually caused by impaired ERE rather than embryonic defects [7]. ERE represents a short period, known as the “window of implantation”, during which the uterine epithelium undergoes extensive structural and functional remodeling to allow embryo attachment [8, 9]. In goats and other ruminants, the receptive state is regulated by progesterone and interferon tau (IFN-τ), which stimulate the expression of adhesion molecules such as SPP1 (secreted phosphoprotein 1, also known as osteopontin) and integrins (ITGB8, αvβ3, and α5β1, etc.). These molecules mediate blastocyst attachment and uterine remodeling [10–12]. Despite its importance, the molecular mechanisms that establish ERE in goats remain poorly understood. Therefore, a deeper understanding of the regulatory mechanisms governing endometrial receptivity will facilitate improvements in reproductive efficiency in goats.

In recent years, large-scale functional genomics programs—such as the Epigenome Roadmap Project, the Farm Animal Genotype-Tissue Expression (FarmGTEx) Project, and the Functional Annotation of Animal Genomes (FAANG) Initiative—have provided valuable resources for studying gene regulation in livestock [13]. These studies have emphasized that epigenetic mechanisms, including chromatin accessibility and histone modifications, play central roles in controlling gene expression [14]. Active chromatin regions contain enhancers and transcription factors (TFs) that collectively establish cell type-specific gene programs, which are dynamically regulated and closely associated with biological processes such as cellular differentiation and development [15, 16]. Enhancers function as *cis-*regulatory elements that interact with target gene promoters through chromatin looping, with their activity modulated by both H3K27ac and TF binding [17, 18]. Among these, super-enhancers (sEnhs)—large clusters of enhancers with high TF occupancy—play pivotal roles in driving the transcriptional activation of genes essential for cell identity and function [19, 20]. Through the recruitment of coactivators such as the Mediator complex and p300, sEnhs maintain an open chromatin architecture and amplify gene expression. Therefore, identifying active sEnhs and associated TFs in goat endometrial tissues is crucial for elucidating the molecular mechanisms underlying endometrial receptivity.

Previous studies have identified several TFs that play critical roles in the establishment of ERE and blastocyst implantation in mammals, including HAND2 [21], FOXO1 [22], and NF-κB [23]. Mouse genetic studies have further revealed that approximately 3% of sEnhs regulate reproductive genes through the enrichment of estrogen receptor α (ERα) binding sites, highlighting the role of hormonal regulation in fertility [24]. In humans, key TFs such as FOS, GATA2/3, MAFK, TFAP2C, and PPARG cooperate with sEnhs to form a core regulatory network that maintains trophoblast identity and drives differentiation [25]. In contrast, no goat ERE-related TFs or *cis*- regulatory elements have been reported.

In this study, we employed an integrative multi-omics strategy to identify TFs and sEnhs involved in the endometrial regulation during the peri-implantation window. This approach revealed how chromatin remodeling and histone acetylation (H3K27ac) coordinate transcriptional activation to establish endometrial receptivity. We defined key TFs, enhancer elements, and sEnh-associated gene networks, highlighting regulators such as *FOSL2*, *KLF6*, *IFI6*, *MCL1*, *SDC4*, and *IL6R* that orchestrate uterine remodeling. Altogether, these findings advance our understanding of the epigenomic mechanisms underlying uterine receptivity in goats and provide a foundation for improving reproductive efficiency in ruminants through targeted epigenetic interventions.

## Materials and methods

### Animals and ethical statement

Six healthy multiparous Dazu black goats were sourced from Tengda Animal Husbandry Co., Ltd. (Chongqing, China) and housed under standardized feeding and management conditions at the Experimental Goat Farm of the Black Goat Research Institute, Southwest University. All experimental procedures were approved by the Animal Ethics Committees of Southwest University (permit number: IACUC-20240506-06) and conducted in accordance with the institutional animal care guidelines.

### Experimental design and sample collection

**Fig. 1:**
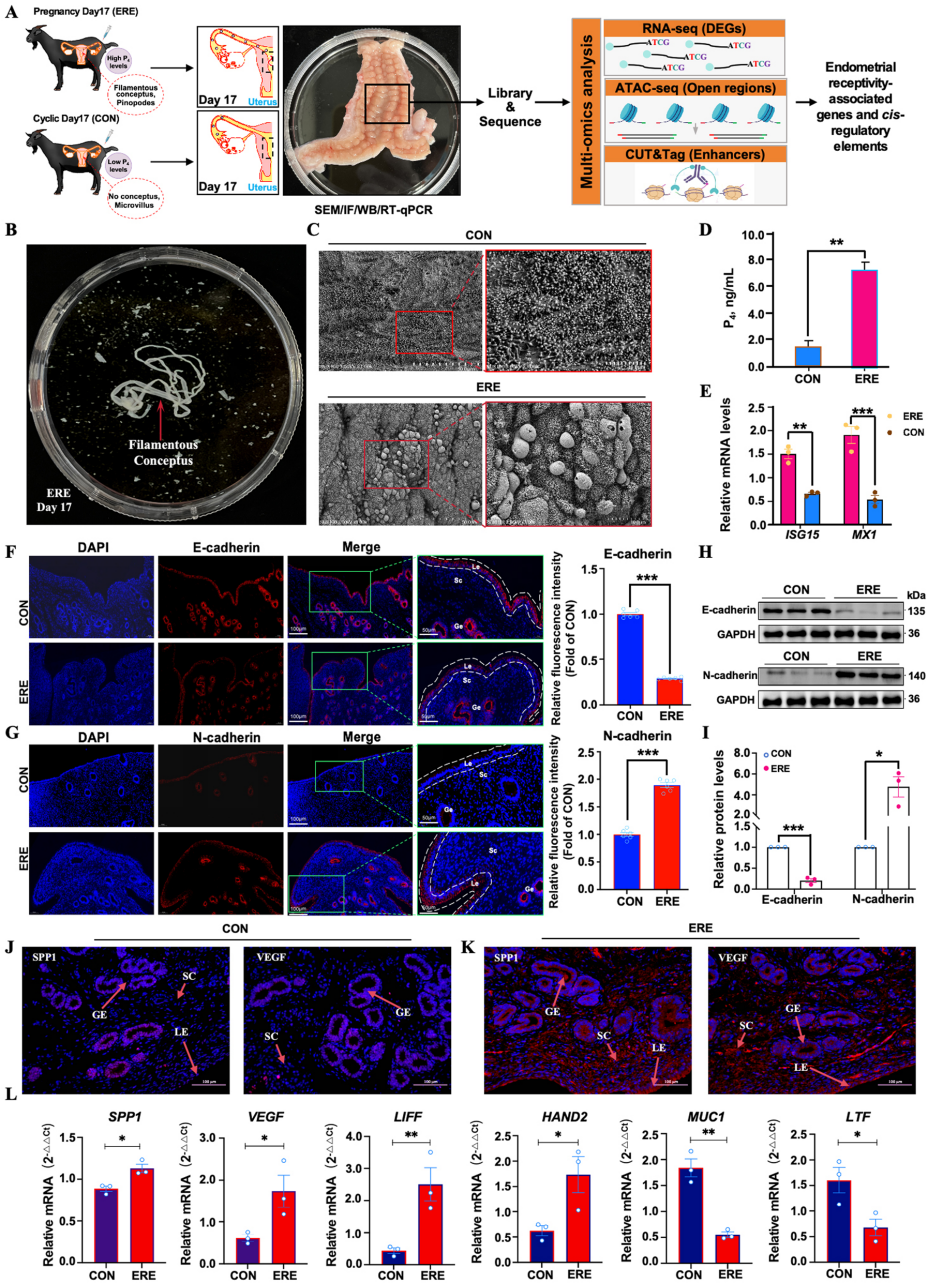
Experimental overview and validation of endometrial receptivity establishment in goats. **A** Schematic representation of the multi-omics workflow integrating RNA-seq, ATAC-seq, and CUT&Tag (H3K27ac) to characterize the chromatin and transcriptional landscapes of the goat endometrium. **B** Filamentous conceptuses observed on pregnancy d 17. **C** Scanning electron microscopy (SEM) showing characteristic microvilli morphology of receptive uterine epithelium. Left scale bar, 50 μm; Right scale bar, 10 μm. **D** and **E** Serum P_4_ levels (**D**) and interferon-stimulated gene (*ISG15*, *MX1*) expression levels (**E**) in CON and ERE group. **F** and** G** Immunofluorescence of E-cadherin (**F**) and N-cadherin (**G**) expression, indicating epithelial transition during receptivity. Left scale bar, 100 μm; Right scale bar, 50 μm. **H** and **I** Immunoblot analysis confirming the protein-level changes of E-cadherin (**H**) and N-cadherin (**I**). **J** and **K** Immunofluorescence of SPP1 (**J**) and VEGF (**K**) expression, showing elevated adhesion and angiogenesis markers in the receptive uterus. Scale bars, 100 μm. **L** RT-qPCR validation of key receptivity markers (*SPP1*, *VEGF*, *LIF*, *HAND2*, *MUC1*, and *LTF*). Values represent mean ± SEM. ^*^P < 0.05, ^**^P < 0.01, ***P < 0.001 Student’s *t*-test

To investigate the chromatin dynamics and transcriptional regulatory underlying endometrial receptivity, we performed RNA-seq, ATAC-seq, and CUT&Tag (H3K27ac) analysis on uterine tissues collected from goats at two physiological stages: receptive and non-receptive (Fig. 1A). The goats with regular estrous cycles were synchronized according to our previous method [26], followed by natural mating with bucks. The day of mating was designated as Day 0 (d 0). On Day 17 (d 17), corresponding to the peri-implantation stage in goats, all animals were euthanized by intravenous injection of sodium barbiturate (30 mg/kg) at the experimental goat farm of Southwest University (Chongqing, China). Three goats ultimately confirmed as non-pregnant were assigned to the non-receptive group (estrous cycle d 17; control, CON; *n* = 3), while the other three goats confirmed pregnant were assigned to the receptive group (pregnancy d 17; endometrial receptivity, ERE; *n* = 3). Uteri were collected and flushed with ice-cold phosphate-buffered saline (PBS) and transported on ice to the laboratory. Each uterus was opened longitudinally. Pregnancy status in the ERE group was confirmed based on the presence of morphologically normal filamentous conceptuses in the uterine flushing. A portion of the endometrial tissues was then rapidly frozen using liquid nitrogen and stored at −80 °C for further analysis. Another portion of the full-thickness uterus was fixed in 4% paraformaldehyde for 24 h and embedded in paraffin for histological analysis.

### Enzyme-linked immunosorbent assay (ELISA)

Serum progesterone (P_4_) concentrations were determined to assess hormonal differences between the non-receptive and receptive groups. Measurements were performed using a commercial goat-specific ELISA kit (Ruixin Bio, China) following the manufacturer’s protocols. Colorimetric detection was performed using TMB substrate, and absorbance was recorded at 450 nm using a microplate reader. Each sample was analyzed in duplicate. Data were processed using GraphPad Prism v10.3.1 (GraphPad Software, San Diego, CA, USA).

### Scanning electron microscope (SEM) analysis

Endometrial tissues were fixed in 2.5% glutaraldehyde overnight at 4 °C, washed with PBS, post-fixed in 1% osmium tetroxide for 1 h, and dehydrated through a graded ethanol series. After treatment with 2% isoamyl alcohol for 3 h, the samples underwent critical point drying with CO_2_. They were then mounted and coated with silver conductive plastic and examined under a Thermo Scientific Phenom scanning electron microscope. Representative micrographs were obtained to evaluate the morphology of the uterine luminal surface.

### Immunofluorescence assay

Fresh endometrial tissues were fixed in 4% fixative solution (FB002, Invitrogen, Waltham, MA, USA) overnight at 4 °C. Samples were then embedded in paraffin and sectioned at 5 μm thickness. After deparaffinization and rehydration, sections were blocked with 1% bovine serum albumin (BSA) for 1 h at room temperature. Primary antibodies were incubated overnight at 4 °C, including anti-SPP1, anti-VEGF, anti-E-cadherin, and anti-N-cadherin (all 1:1,000, Bioworld, Nanjing, China). After washing, sections were incubated with appropriate secondary antibodies and counterstained with Hoechst 33342 (1:1,000, Beyotime, Shanghai, China). Images were acquired using a Leica TCS SP8 confocal microscope (Wetzlar, Germany). Fluorescence intensity was quantified using Case Viewer software (C.V.2.4, Digihail Electronic, Jinan, China).

### Western blotting

Total protein from endometrial tissues was extracted using RIPA buffer (Beyotime, Shanghai, China) and quantified with a BCA protein assay kit (Beyotime, Shanghai, China). Equal amounts of protein were separated by 12% SDS-PAGE (Bio-Rad, USA) and transferred to PVDF membranes. Membranes were blocked with 5% non-fat milk for 1 h at room temperature and then incubated overnight at 4 °C with primary antibodies against E-cadherin and N-cadherin (1:1,000; Bioworld, Nanjing, China). After washing with TBST, membranes were incubated with HRP-conjugated secondary antibodies (1:1,000, Beyotime, Shanghai, China) for 2 h at room temperature. Protein bands were visualized using the Omni-EC™ Femto Light Chemiluminescence kit (EpiZyme, Shanghai, China) and imaged with a Bio-Rad chemiluminescence detection system. Band intensities were quantified using ImageJ software (v6.0; NIH, Bethesda, MD, USA).

### RNA extraction and real-time quantitative PCR

RNA extractions were performed as previously described [27]. Briefly, total mRNA was extracted using TRIzol reagent (Invitrogen, CA, USA). RNA purity was assessed by OD_260_/OD_280_ ratios using a NanoDrop 2000 spectrophotometer (NanoDrop Technologies, Wilmington, DE, USA), and integrity was verified using a Bioanalyzer 2100 system (Agilent Technologies, CA, USA). Complementary DNA (cDNA) was synthesized using the PrimeScript™ RT regent kit (TaKaRa, Otsu, Japan) according to the manufacturer’s instructions. RT-qPCR reactions were performed on a QuantStudio^®^ 3 system (Applied Biosystems, Foster City, CA, USA) using TB Green^®^ Premix Ex Taq™ II (Takara, Otsu, Japan). Each sample was analyzed in triplicate. Primers were synthesized by Sangon Biotech (Shanghai, China) and are listed in Table S1. The housekeeping gene *GAPDH* served as the internal control, and relative expression levels were calculated using the 2^−ΔΔCt^ method [28].

### RNA-seq library construction and sequencing

High-quality total RNA was used to construct RNA-seq libraries on the MGI high-throughput sequencing platform (Frasergen, Wuhan, China). All library preparations were performed according to standard protocols, which included poly(A) mRNA enrichment, fragmentation, cDNA synthesis, adapter ligation, and PCR amplification. Library quality and concentration were confirmed using a Qubit™ fluorometer and Agilent 2100 Bioanalyzer. Clean reads were aligned to the goat reference genome (ARS1.2) using HISAT2 (v2.2.1). Gene-level read counts were calculated using RSEM (v1.3.3), and FPKM values were obtained after normalization for sequencing depth and transcript length. Differentially expressed genes (DEGs) were identified using DESeq2 (v1.30.0) with selection criteria of |log_2_Fold Change| > 1 and false discovery rate (FDR) < 0.05. Functional enrichment of DEGs was performed through Gene Ontology (GO) and Kyoto Encyclopedia of Genes and Genomes (KEGG) analyses using the Metascape platform (https://metascape.org) with *P* < 0.05 as the significance threshold.

### ATAC-seq library construction and sequencing

The assay for transposase-accessible chromatin with sequencing (ATAC-seq) was performed as previously described [29] using the TruePrep™ DNA Library Prep kit (Frasergen, Wuhan, China). Approximately 5 mg of frozen endometrial tissue was homogenized in ice-cold PBS, and nuclei were isolated for tagging using Tn5 transposase. Following purification and PCR amplification, libraries were sequenced on an Illumina NovaSeq platform (San Diego, CA, USA) in paired-end 150 bp mode (PE150).

### Differential accessibility and footprinting analyses

After quality filtering with Trimmomatic, clean ATAC-seq reads were aligned to the goat reference genome (ARS1.2) using Bowtie2. Peaks were called using MACS3, and differential accessible regions (DARs) were identified with the DiffBind package (v3.4.0) based on the DESeq2 (v1.36.0) model. Transcription factor footprinting analysis was performed using HINT-ATAC, and motif enrichment was determined using the MEME (Multiple Em for Motif Elicitation) Suite. ATAC-seq signal profiles were visualized with ggplot2, plotting average accessibility across gene bodies with 3 kb flanking regions (upstream and downstream). Correlation between chromatin accessibility and transcriptional activity was assessed by overlapping promoter-associated DARs with DEGs. Signal visualization and genome browser tracks where genome browser tracks were generated using Integrative Genomics Viewer (IGV, v2.18.2).

### CUT&Tag library construction and sequencing

Cleavage under targets and tagmentation (CUT&Tag) was performed using flash-frozen endometrial tissues, following the same nuclei isolation procedure as for ATAC‑seq. Briefly, nuclei were bound to concanavalin A-coated magnetic beads (BP531; BioMag Plus) and incubated for 10 min at room temperature. Samples were then incubated with primary antibody (anti-H3K27AC) for 1 h at room temperature. After washing to remove unbound antibodies, nuclei were incubated with secondary antibody (goat anti-rabbit IgG; ab6702; Abcam) for 1 h at room temperature, followed by three washes with DIG buffer. Targeted tagmentation was carried out using the protein G-Tn5 transposome. After 1 h incubation, unbound complexes were removed by washing with 1 × Dig‑300 buffer. Bound DNA fragments were purified using phenol-chloroform extraction and AMPure XP beads (Beckman Coulter). Libraries were PCR-amplified and sequenced on an Illumina NovaSeq platform (Frasergen, Wuhan, China). Clean reads were processed similarly to ATAC-seq data—aligned to the goat reference genome (ARS1.2) with Bowtie2, peak calling with MACS3, and visualization using the WashU Epigenome Browser.

### Prediction of super-enhancers and transcription factors

Super-enhancers were identified using the ROSE (Rank Ordering of Super-Enhancers) algorithm based on H3K27ac enrichment, following the pipeline developed by the Young Laboratory [30, 31]. The algorithm ranked enhancers by signal intensity and clustering of H3K27ac peaks to distinguish typical enhancers from sEnhs. To identify TFs associated with these sEnhs, motif scanning was performed using FIMO (Find Individual Motif Occurrences; v5.4.1) within the MEME Suite [32, 33]. Default parameters were used to detect enriched motifs within sEnh regions.

### ATAC-seq and CUT&Tag data analysis

Raw paired-end reads were filtered using Trimmomatic (v0.39) to remove adapters and low-quality bases. Quality control was performed using FastQC (v0.11.9). Clean reads were aligned to the goat reference genome (ARS1.2) using Bowtie2 (v2.3.5). Low-quality alignments, PCR duplicates, and organelle reads were removed using Samtools (v1.12) and Picard (v2.25.6). Valid read pairs were retained for downstream analysis. Peaks were called using MACS3 (v3.0.0a6), and reproducibility among biological replicates was assessed by comparing overlapping peak regions. Signal enrichment near transcription start site (TSS ± 3 kb) and across gene bodies was visualized with deepTools (v3.5.1). Gene annotation of peak regions was performed using ChIPseeker [34], and genome-wide enrichment was visualized using Gviz and the WashU Epigenome Browser (http://epigenomegateway.wustl.edu/browser/). Correlations among RNA-seq, ATAC-seq, and CUT&Tag datasets were evaluated using Pearson’s correlation coefficients.

### Differential peak analysis

Peak annotation for ATAC-seq and H3K27ac CUT&Tag data was performed using ChIPseeker, based on proximity to TSS. Functional annotation and pathway enrichment analyses were conducted using clusterProfiler, integrating GO and KEGG databases. Motif enrichment within peak regions was determined using the MEME Suite (v5.4.1). Signal intensity across gene bodies (± 3 kb flanking regions) was calculated using custom R scripts. Peaks from biological replicates were merged to create consensus peak sets for comparison. DARs from ATAC-seq and differential H3K27ac peaks (DPs) from CUT&Tag were identified using DiffBind, with thresholds of |log_2_FoldChange| > 1 for DARs or > 0.58 for DPs and FDR < 0.05. Associated genes were annotated using ChIPseeker, and enriched biological functions were determined via clusterProfiler. Motif analysis of DARs and DPs was conducted with the MEME suite, and TF binding site variations were further analyzed using HINT-ATAC [35] and validated against the JASPAR database.

## Results

### Phenotypic identification of endometrial receptivity in the goat

To verify the establishment of endometrial receptivity (ERE), we examined uterine morphological and key molecular markers. Filamentous conceptuses were observed in the ERE group, confirming successful implantation (Fig. 1B). SEM analysis revealed dense microvilli on the luminal epithelial of the CON group, whereas the ERE group showed smooth surfaces with pinopodes—features typical of receptive endometrium (Fig. 1C). Serum P_4_ levels were significantly higher in the ERE group (Fig. 1D). This difference, specifically observed at Day 17, reflects the expected physiological divergence between receptive and non-receptive uteri. Since no goat-specific ELISA is available for IFN-τ, we indirectly assessed embryonic IFN-τ activity by measuring the interferon-stimulated genes *ISG15* and *MX1*, both known IFN-τ targets in ruminants [36, 37]. Their expression was markedly upregulated in ERE endometria (*P* < 0.01, Fig. 1E), confirming active conceptus-derived IFN-τ signaling. Plasma membrane transformation (PMT) was evaluated using immunofluorescence for E-cadherin and N-cadherin. E-cadherin localization shifted from continuous lateral membranes in CON tissues to a more apical and discontinuous pattern in ERE endometria (Fig. 1F and G), consistent with transient epithelial remodeling that facilitates blastocyst adhesion [38–40]. Western blot further confirmed decreased E-cadherin and increased N-cadherin expression in ERE tissues (Fig. 1H and I, Fig. S1).

Representative genes affected the endometrial receptivity and implantation (e.g., *SPP1*, *VEGF*, *LIF*, *HAND2*, *MUC1*, and *LTF*) were selected based on previous reports demonstrating their conserved roles in regulating epithelial remodeling, embryo adhesion, and uterine receptivity establishment in ruminants. The receptivity-related genes (*SPP1*, *VEGF*, *LIF*, *HAND2*) were upregulated, while anti-receptivity markers (*MUC1*, *LTF*) were downregulated (Fig. 1L). Immunofluorescence confirmed higher expression of SPP1 and VEGF in receptive uteri (Fig. 1J and K). Together, these morphological and molecular data confirm that d 17 of pregnancy represents the receptive phase in goats.

### Overview of multi-omics datasets

To explore the regulatory basis of ERE, we generated integrated datasets using RNA‐seq, ATAC‐seq, and H3K27ac CUT&Tag. Each RNA-seq library yielded ~ 0.23 billion clean reads, with > 95% uniquely mapped (Table S2). ATAC‐seq and CUT&Tag libraries each produced ~ 0.5 billion paired reads per sample, with an average mapping rate of 98.9% (Table S3). Quality metrics, including Non-Redundant Fraction (NRF) and PBC values, confirmed high data complexity and reproducibility, ensuring suitability for downstream epigenomic analysis.

### Identification of genes associated with endometrial receptivity

**Fig. 2:**
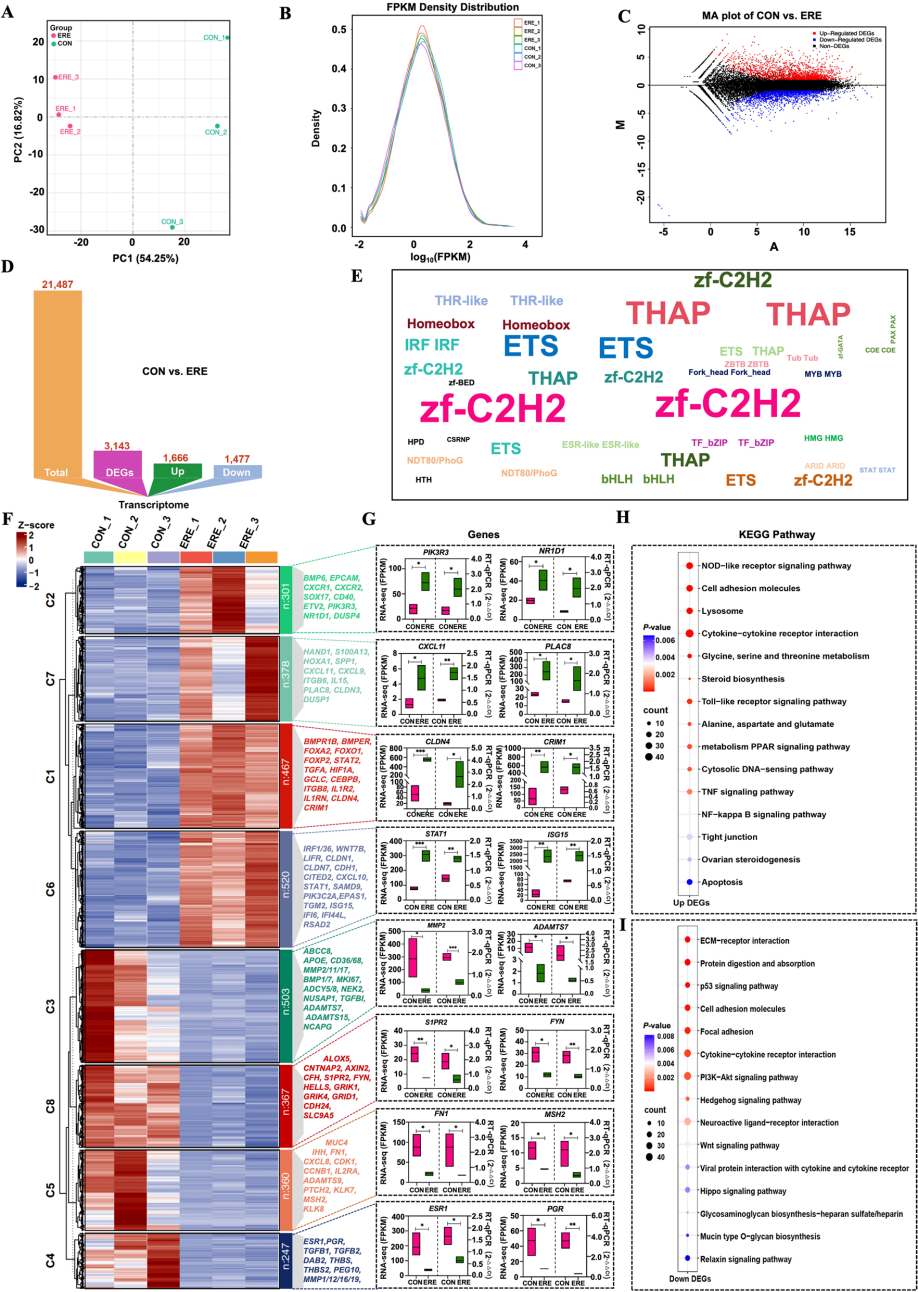
Transcriptomic profiling reveals key regulatory signatures of endometrial receptivity. **A** PCA showing the first principal component (PC1) against the second principal component (PC2) of different receptive uterine status in goats. **B** Gene expression abundance distribution map. **C** and **D** Differentially expressed genes (DEGs) identified between groups. **E** and **F** TF family enrichment (**E**) and K-means clustering (**F**) highlighting co-regulated transcriptional programs driving receptivity. **G** RT-qPCR validation of representative DEGs corroborating RNA-seq data. Data shown as mean ± SEM of 3 biological replicates. **H** and **I** KEGG analysis indicating that up- (**H**) and down-regulated (**I**) DEGs are enriched in pathways related to ERE establishment. ^*^P < 0.05, ^**^P < 0.01, ***P < 0.001

Principal component analysis showed clear transcriptomic separation between CON and ERE samples (Fig. 2A). A total of 21,467 transcripts were detected, and the gene expression abundance distribution diagram is shown in Fig. 2B. Further differential analysis identified 3,143 DEGs (1,666 upregulated and 1,477 downregulated) in ERE (Fig. 2C and D, Table S4). Enriched TF families indicated zf-C2H2, ETS, THAP, IRF, and Homeobox (Fig. 2E, Table S5). K-means clustering grouped DEGs into eight co-expression modules showing coordinated activation of immune, adhesion, and differentiation pathways (e.g., JAK-STAT, MAPK, NF-κB, PI3K-Akt, and AMPK signaling pathways) and suppression of estrogen- and proliferation-related signaling (e.g., Wnt, mTOR, estrogen, and cell cycle signaling pathways) (Fig. 2F–I, Table S6–S7). Notably, key upregulated genes—*SPP1*, *FOXO1*, *DUSP4*, *ISG15*, *MX1*, and *ITGB8*—were linked to receptivity, whereas *ESR1*, *PGR*, *BMP1/7*, *MMP1/12/16*, *AXIN2*, and *FYN* were repressed, reflecting the hormonal adaptation for implantation. Moreover, genes involved in implantation, progesterone responsiveness, extracellular matrix remodeling, maternal–fetal communication, extracellular matrix remodeling, and nutrient transport (e.g., *HIF1A*, *RUFY4*, *FOXA2*, *FOS*, *HAND1*, *PLAC8*, *CDH1*, and *FOSL2*) were also activated, suggesting coordinated regulation of tissue remodeling and immune tolerance during the receptive phase. RT-qPCR of 16 representative genes confirmed RNA-seq accuracy (Fig. 2G). These transcriptomic results reveal a shift from proliferative to adhesive and immune-tolerant endometrial states, characteristic of receptivity.

### Chromatin remodeling during receptivity establishment

**Fig. 3:**
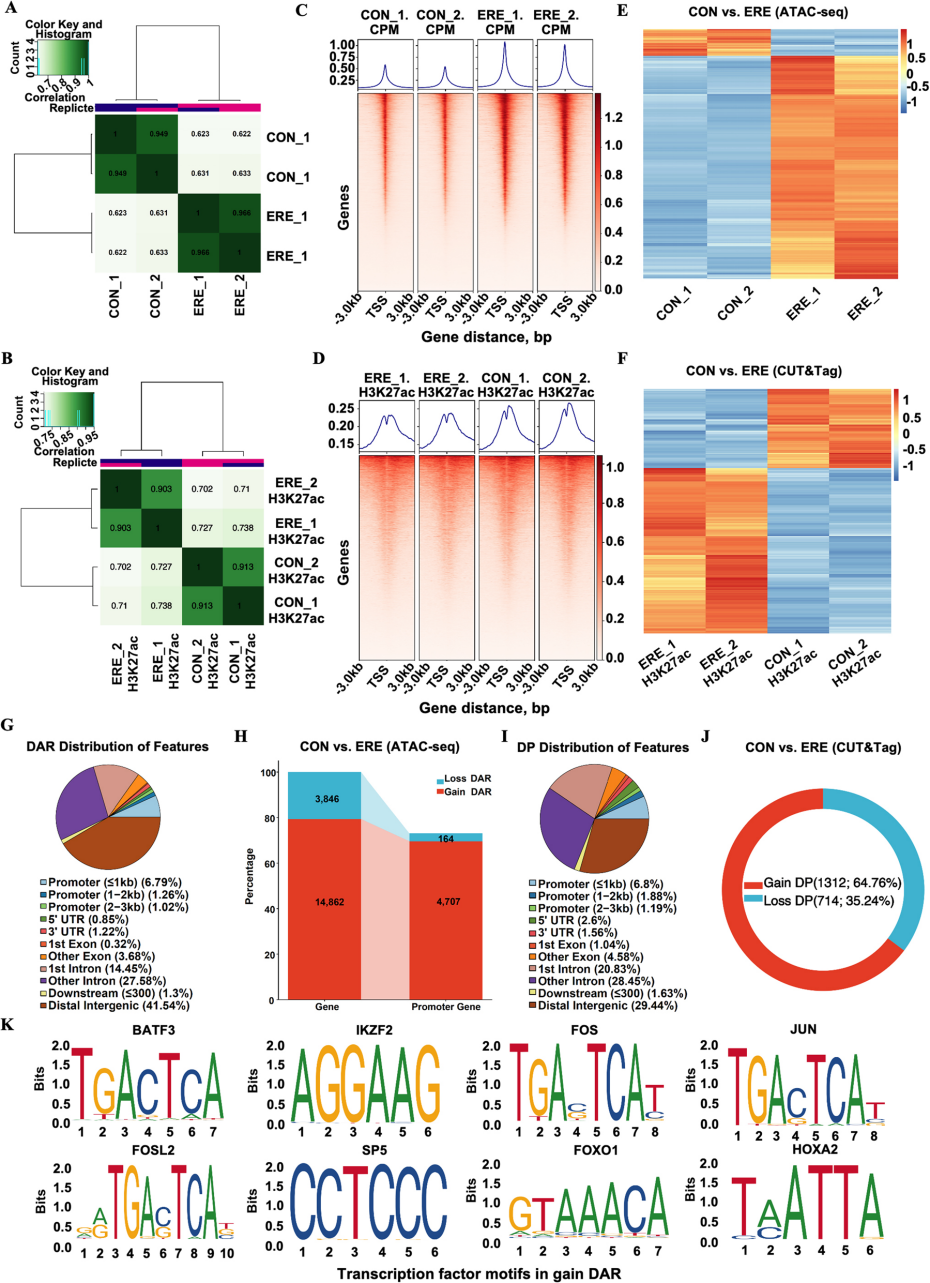
Chromatin accessibility and H3K27ac landscapes define epigenetic remodeling of the receptive endometrium. **A** and **B** Correlation heatmaps confirming high reproducibility among replicates. **C** and **D** Aggregated ATAC-seq (**C**) and H3K27ac signals (**D**) around TSSs, validating data quality and promoter activity. **E** and **F** Clustering of DARs (**E**) and DPs (**F**), revealing distinct epigenetic profiles between receptive and non-receptive groups. **G** and **I** Genomic distribution of DARs (**G**) and DPs (**I**). **H** and **J** Annotation of genes associated with DARs (**H**) and DPs (**J**), linking epigenetic changes to potential gene targets. **K** Motif analysis identifying enriched TF-binding motifs in DARs

To explore the epigenetic regulation underlying these transcriptional changes, we profiled chromatin accessibility and histone acetylation (H3K27ac) using ATAC-seq and CUT&Tag. H3K27ac marks both promoters and distal enhancers [41]. Irreproducible discovery rate (IDR) (Fig. S2A and C) and correlation heatmaps showed high reproducibility between biological replicates (Fig. 3A and B), with strong enrichment of signals near TSSs (Fig. 3C and D). Accessible regions and H3K27ac peaks were predominantly located within 3 kb of transcription start sites, consistent with promoter and enhancer activation. In total, 64,966 DARs (57,991 gained and 6,975 lost) and 4,043 DPs (2,733 gained and 1,310 lost) were detected (Fig. S2B and D), indicating broad epigenomic reprogramming during receptivity establishment (Fig. 3E and F). Genomic annotation revealed that DARs were predominantly distributed in intronic (42.03%) and distal intergenic regions—enhancer loci (41.54%), which often harbor potential enhancer elements. A smaller proportion was in promoter regions (9.07%), exons (4.0%), UTRs (2.07%), or within 300 kb downstream of the transcription start site (1.3%) (Fig. 3G). Similar distributions were observed for both gained and lost peaks (Fig. S2E and F), as well as for H3K27ac CUT&Tag signals (29.44% intergenic regions, 49.28% intron, 9.87% promoter, 5.62% exons, 4.16% UTRs, and 1.63% were less than 300 kb region downstream of the TSS) (Fig. 3I; Fig. S2G and H), indicating a consistent epigenomic remodeling pattern across regulatory layers during establishment of ERE.

### Genomic chromatin accessibility, H3K27ac profiling, and targeted gene analysis

To elucidate the functional consequences of chromatin remodeling during the establishment of ERE, genes associated with DARs, and H3K27ac DPs were annotated based on proximity to TSSs, respectively. In total, 14,150 genes linked to gained DARs and 3,562 linked to lost DARs, with promoter-associated DARs showing the strongest regulatory potential (Fig. 3H, Tables S8–S9). Pathway enrichment linked promoter-associated DARs with tight junction, cell adhesion, focal adhesion, immune regulation, AMPK, MAPK, FoxO, VEGF, and autophagy signaling pathways (Fig. S3A, Table S10). Conversely, peaks with reduced accessibility were enriched in cytokine-cytokine receptor interaction and cell adhesion molecules signaling (Table S11). Similarly, analysis of H3K27ac CUT&Tag data revealed 1,271 genes associated with gained DPs and 708 with lost DPs (Fig. 3J, Tables S12–S13). Pathway enrichment linked gaining H3K27ac signals with promoting epithelial remodeling, stress response, and cell survival (e.g., PI3K-Akt, MAPK, and p53 signaling), whereas those losing acetylation were involved in estrogen and TGF-β signaling (Fig. S3B, Table S14). Together, these results demonstrate that ERE involves chromatin activation at genes driving adhesion, immune tolerance, and metabolic adaptation, while estrogen and the cell cycle are selectively silenced to prepare the uterus for implantation.

### Identification of key transcription factors in the establishment of endometrial receptivity

**Fig. 4:**
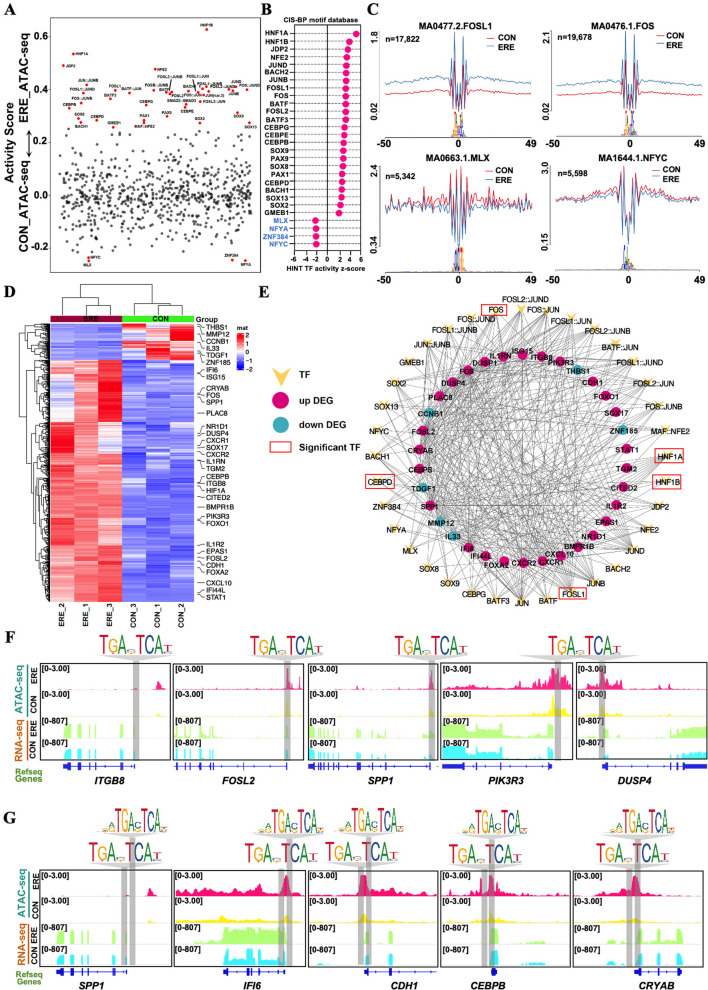
Regions of differential chromatin accessibility are enriched for key transcriptional regulators. **A** HINT-ATAC footprinting identifies TFs with altered binding activity in accessible chromatin. **B** Differential TF activity analysis highlights specific activation of FOS, FOSL2, HNF1A, and HNF1B, etc., in the receptive endometrium. **C** Nucleotide-resolution footprints confirm active TF binding at these motifs. **D** and **E** Expression heatmap (**D**) and TF-gene network (**E**) reveal core transcriptional circuits controlling receptivity. **F** and **G** Presence of FOS and FOSL2 motifs in open chromatin regions of critical receptivity genes

To accurately identify potential candidate TFs related to the establishment of ERE, we performed a joint analysis of the motif enrichment and HINT-ATAC. First, motif enrichment analysis revealed the TFs with gained and lost peaks (Fig. 3K, Table S15), which provided further insights into the transcriptional regulatory landscape. Next, to resolve TF activity dynamics, HINT-ATAC footprinting analysis identified a subset of activated TFs in receptivity endometria that play central roles in implantation-associated processes. Activated TFs included AP-1 complex members (FOSL2, FOS, JUN, BATF, and BATF3), HNF1A/B, CEBP family (CEBPD/E/G), SOX8/9/13, and homeobox proteins (MSX and HOX families) (Fig. 4A–C, Tables S16–S17). Repressed TFs such as MLX and NFYA were associated with cell-cycle and metabolic regulators. Integrating footprinting with RNA-seq identified TF-target networks involving *SPP1*, *ITGB8*, *PLAC8*, *DUSP4*, *PIK3R3*, *NR1D1*, *FOXO1*, *IFI6*, *ISG15*, *EPAS1*, *HIF1A*, *CDH1*, and *BMPR1B*, related to cytokine signaling, extracellular matrix remodeling, and hypoxia response (Fig. 4D and E, Table S20). Notably, FOS directly bound and potentially activated *ITGB8*, *FOSL2*, *PIK3R3*, and *DUSP4*, while the FOS-FOSL2 complex jointly regulated *SPP1*, *IFI6*, *CDH1*, *CEBPB*, and *CRYAB* (Fig. 4F and G), highlighting AP-1 complex as a central coordinator of immune and structural transitions in the receptive uterus.

### Chromatin dynamics coordinate with transcriptional profile

**Fig. 5:**
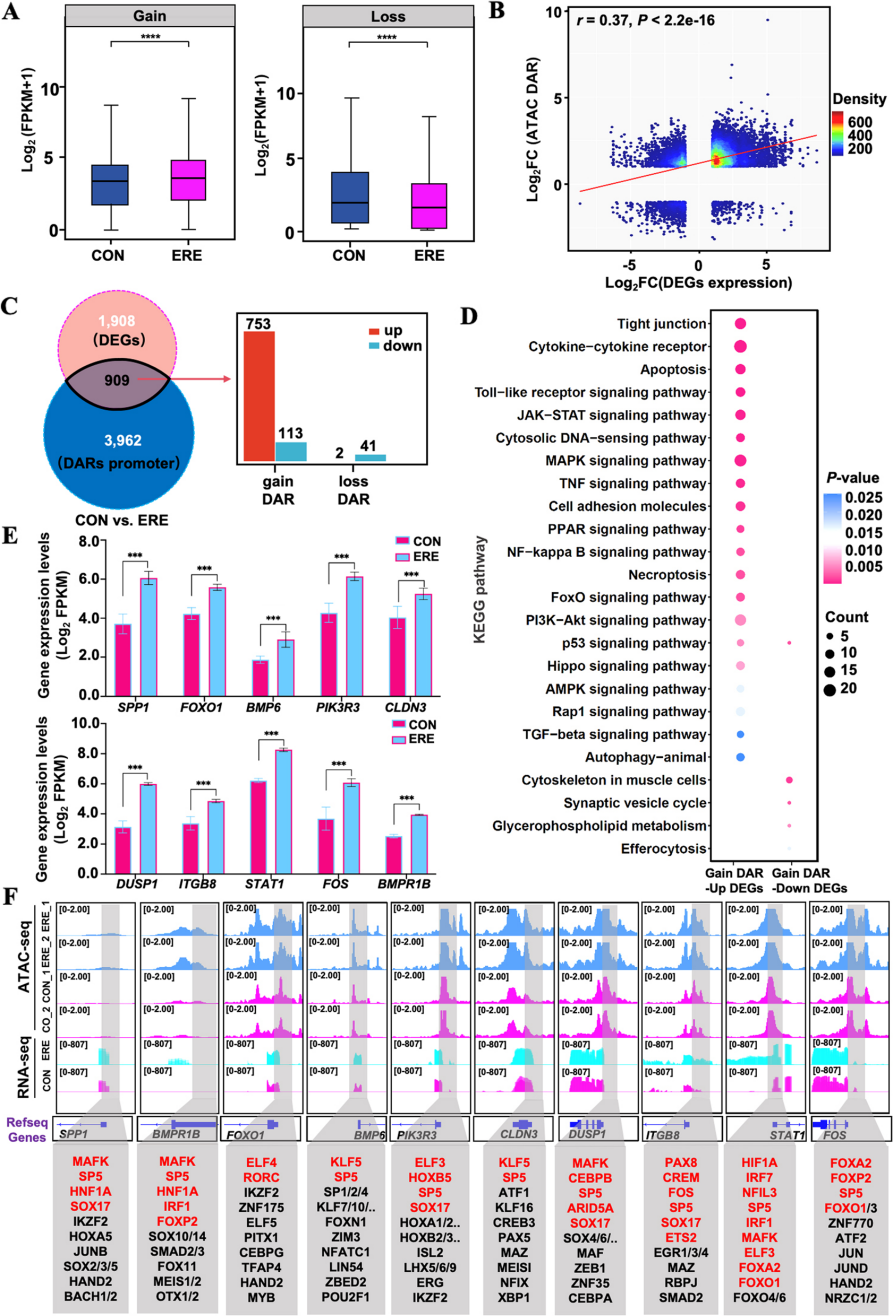
Integration of chromatin dynamics and transcription reveals a direct regulatory target of receptivity. **A–C** Combined analysis of DARs and EDGs identifies genes directly regulated by promoter accessibility. **D** and **E** KEGG analysis (**D**) and RNA-seq (**E**) validation highlight key genes (*SPP1*, *FOXO1*, *BMP6*, *PIK3R3*, *CLDN3*, *DUSP1*, etc.). Error bars = SEM of 3 biological replicates; ***P < 0.001, ****P < 0.0001. **F** Genome browser tracks demonstrate coordinated chromatin opening and gene (*SPP1*, *FOXO1*, *BMP6*, *PIK3R3*, *CLDN3*, *DUSP1*, etc.) activation. Grey boxes indicate intergenic accessible regions and transcription factor footprints identified within those regions

Gene expression is tightly regulated through interaction between chromatin structure and transcription [42]. To explore how chromatin remodeling contributes to ERE, we integrated ATAC-seq and RNA-seq datasets. Genes located within regions of increased chromatin accessibility (gained DARs) showed markedly higher expression in ERE compared with CON endometria (Fig. 5A), consistent with global transcriptomic activation. Correlation analysis confirmed a positive association between chromatin openness and gene expression (*r* = 0.37; Fig. 5B, Table S16), demonstrating that transcriptional activation is closely coupled with chromatin remodeling. Integrative analysis identified 909 DEGs overlapping with promoter-associated DARs, of which 753 genes exhibited both increased accessibility and elevated mRNA levels (Fig. 5C, Tables S17–S18). These genes were enriched in biological processes central to receptivity establishment, including cell adhesion, immune modulation, and uterine remodeling. Representative genes such as *SPP1*, *FOXO1*, *BMP6*, *PIK3R3*, *CLDN3*, *DUSP1*, *ITGB8*, *STAT1*, and *FOS* exhibited coordinated transcriptional upregulation and promoter accessibility (Fig. 5D and E, Table S18 and Table S4). Interestingly, a small subset of 113 genes displayed increased promoter accessibility but reduced expression, suggesting that chromatin opening alone is insufficient for transcriptional activation and may involve additional regulatory mechanisms such as histone methylation or transcriptional repressors (Table S18). Motif and footprinting analyses revealed key regulators (FOXO1, HNF1A, SOX17, and AP-1 family members JUN, JUNB, JUND) occupying these accessible promoters (Fig. 5F, Table S19). Collectively, these findings indicated that ERE establishment is driven by coordinated chromatin remodeling and TF-mediated activation, promoting adhesion, immune tolerance, and structural transformation of the uterus to support blastocyst implantation.

### Genome-wide profiling of H3K27ac-marked enhancers

**Fig. 6:**
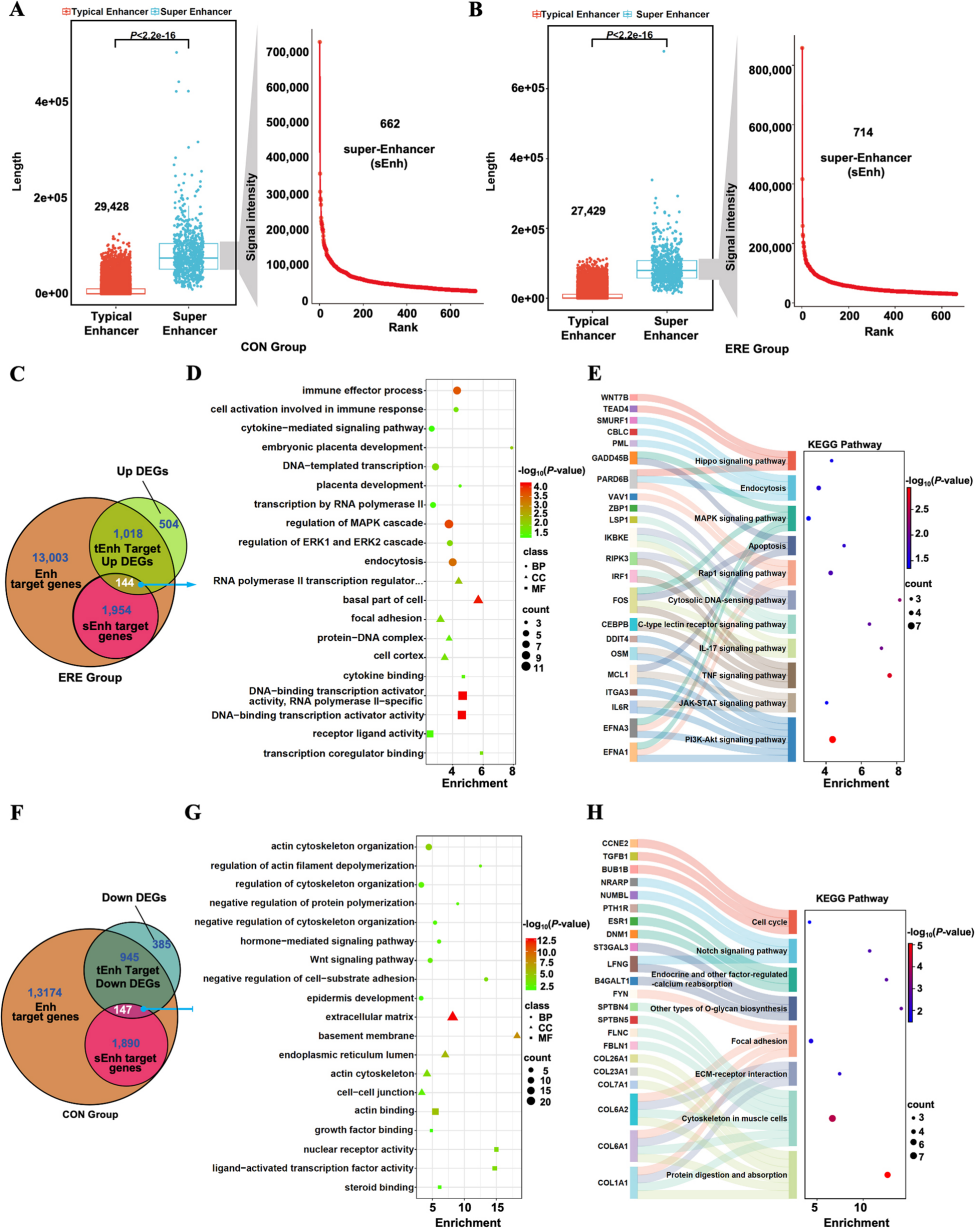
Genome-wide enhancer profiling reveals activation of super-enhancer during receptivity. **A** and **B** Comparative H3K27ac profiling identifies dynamic activation of tEnhs and sEnhs. **C–E** Overlap analysis (**C**), GO enrichment (**D**), and KEGG pathway analysis (**E**) of sEnh-targeted upregulated genes in the ERE group. **F–H** Overlap analysis (**F**), GO enrichment (**G**), and KEGG pathway analysis (**H**) of sEnh-targeted downregulated genes in the CON group

Enhancers are pivotal *cis*-regulatory elements that control tissue-specific transcriptional programs [43]. To characterize enhancer (Enh) activity during receptivity establishment, we profiled H3K27ac modifications using CUT&Tag in CON and ERE endometrial. We identified 30,090 active enhancers in CON and 28,143 in ERE tissues, with distinct patterns corresponding to the transition from a non-receptive to a receptive state (Fig. 6A and B, Table S21). Among these, sEnhs—clusters of densely enhancers with high TF occupancy—were detected in both conditions, 662 in CON and 714 in ERE (Fig. 6A and B, Table S21). Integration with RNA-seq revealed strong positive correlations between sEnh activity and transcriptional upregulation. In ERE tissues, 144 sEnh-linked upregulated DEGs were enriched in pathways associated with immune regulation, placental development, and cell communication, whereas 147 downregulated DEGs in CON tissues were mainly related to cell proliferation and cytoskeletal organization (Fig. 6C–H, Tables S22–S24).

Functional enrichment further showed that ERE-specific sEnh targets were involved in PI3K-Akt, JAK-STAT, MAPK, IL-17, and HIF-1 signaling pathways—crucial for uterine remodeling, placenta development, and maternal–fetal immune tolerance. Genes associated with RNA polymerase II, DNA-binding transcription activator activity, and co-regulator recruitment were also significantly enriched (Fig. 6D and E, Tables S23–S24). Conversely, sEnh-related genes in CON endometria were enriched in cell cycle, Wnt, and focal adhesion pathways, indicating a suppression of proliferative programs during the receptive phase (Fig. 6G and H, Table S23–S24).

Altogether, these data highlight extensive enhancer reprogramming during receptivity establishment, characterized by the activation of immune- and adhesion-related sEnhs and the downregulation of proliferative networks. Dynamic remodeling of H3K27ac-marked enhancers thus emerges as a key mechanism orchestrating uterine readiness for implantation.

### H3K27ac-associated enhancers drive transcription activation

**Fig. 7:**
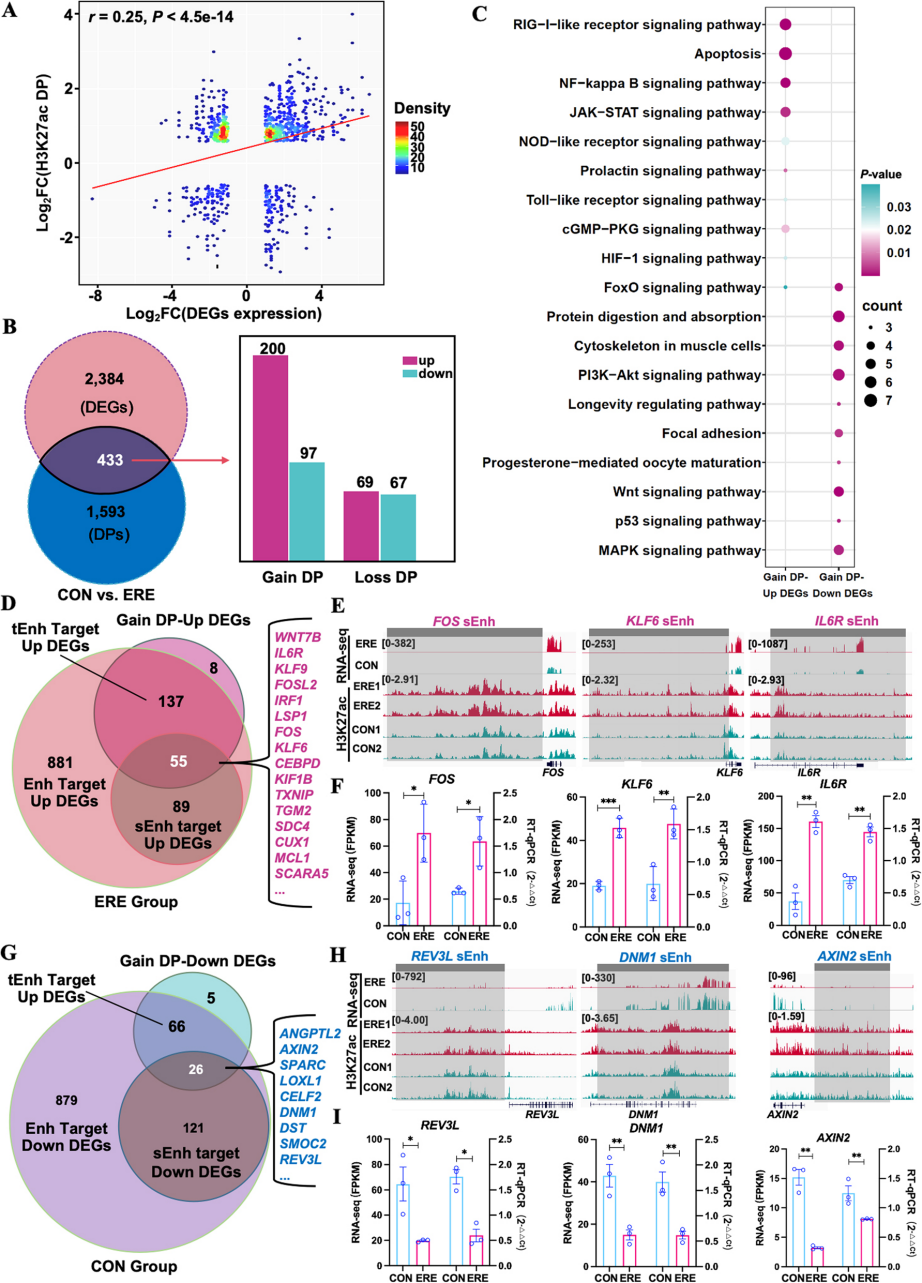
H3K27ac enrichment correlates with transcriptional activation of core receptivity genes. **A–C** Integration of DPs and DEGs reveals a positive correlation and functional enrichment in signaling pathways essential for receptivity. **D** and **E** Overlap between sEnh-targeted and H3K27ac-enriched genes highlights multi-layered epigenetic activation. **F** RT-qPCR validation key activated genes in the ERE group. **G–I** Parallel analysis for sEnh-target DEGs in the CON group. ^*^P < 0.05, ^**^P < 0.01, ^***^P < 0.001

To further determine how H3K27ac modifications influence gene expression, we analyzed differential H3K27ac peaks (DPs) between ERE and CON groups. Genes marked by H3K27ac exhibited significantly higher transcription in the ERE group, consistent with increased chromatin accessibility (Fig. S4, Fig. 4A). This suggests that H3K27ac-mediated chromatin activation contributes to transcriptional upregulation during ERE establishment. Correlation analysis further revealed a positive relationship between H3K27ac intensity and transcript levels (*r* = 0.25; Fig. 7A, Table S25). Among 433 DEGs overlapping DPs, 200 showed both increased H3K27ac and elevated mRNA expression (Fig. 7B, Table S26). These genes were enriched in apoptosis, NF-κB, JAK-STA, and FoxO signaling pathways—central to epithelial remodeling and immune signaling—whereas downregulated genes were mainly involved in PI3K-Akt, Wnt, and MAPK signaling, consistent with reduced proliferative activity (Fig. 7C, Table S27). Integration of DPs with enhancer annotations revealed that numerous DEGs were controlled by tEnhs or sEnhs (Fig. 7D and G, Table S28). RT-qPCR validation confirmed significant upregulation of *FOS*, *KLF6*, and *IL6R* in ERE tissues and higher expression of *REV3L*, *DNM1*, and *AXIN2* in CON tissues (Fig. 7E–I; *P* < 0.01, Student’s *t*-test). These findings demonstrate that H3K27ac-mediated enhancer acetylation is a major driver of transcriptional programs required for uterine receptivity, regulating genes involved in differentiation, adhesion, and immune adaptation.

### Super-enhancer-based transcriptional network is defined in the receptive state.

**Fig. 8:**
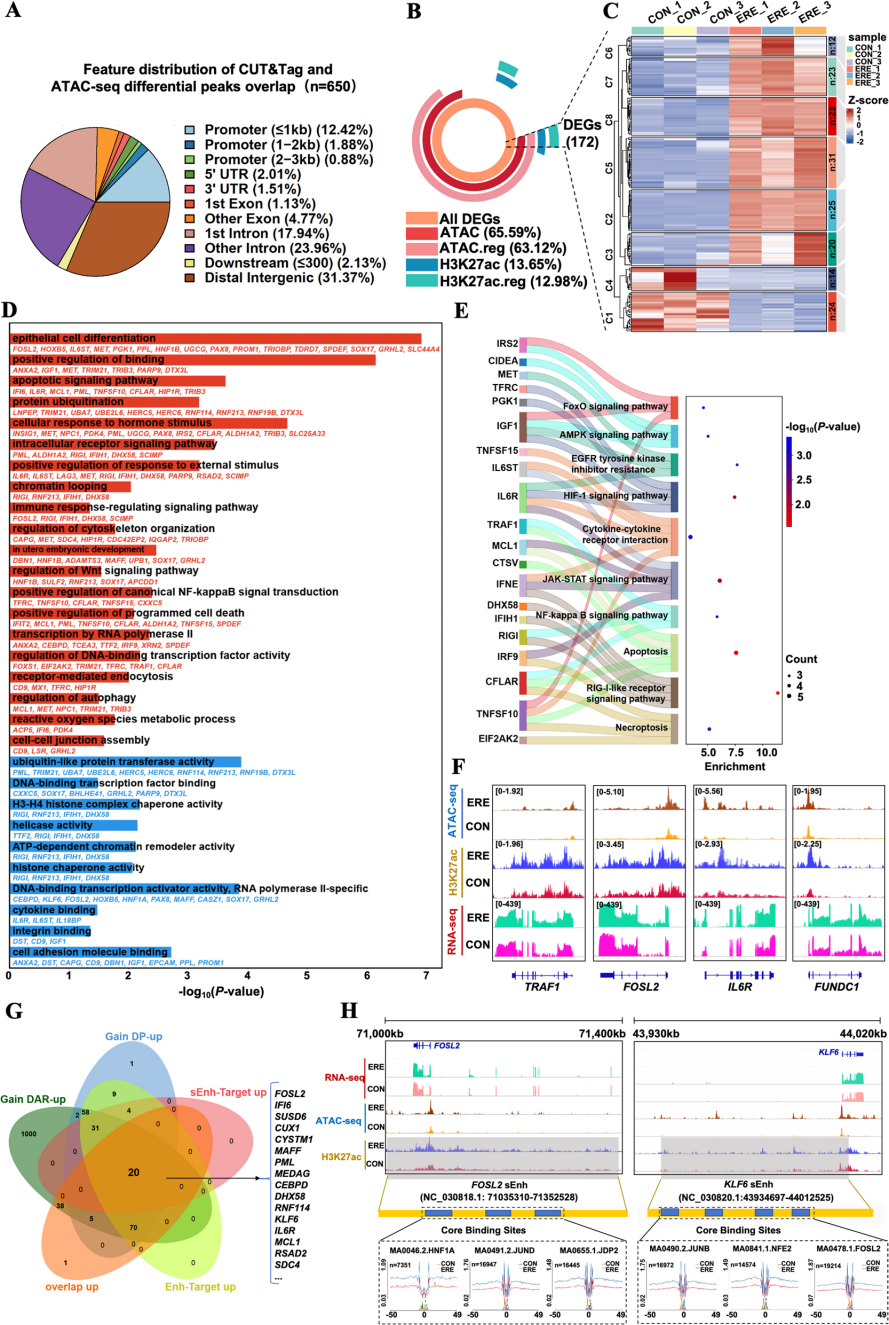
Multi-omics integration identifies key transcriptional regulatory networks driving endometrial receptivity. **A** Integration of DARs, DPs, and DEGs identifies 650 high-confidence epigenetic targets. **B** Proportion of DEGs associated with chromatin accessibility and/or H3K27ac changes, underscoring the predominant role of epigenomic reprogramming. **C** Expression heatmap of the 172 DEGs co-regulated by both mechanisms. **D** and **E** GO (**D**) and KEGG (**E**) enrichment analysis reveal pathways associated with ERE establishment. **F** Integrated epigenomic and transcriptional tracks at representative core genes (*TRAF1*, *FOSL2*, *IL6R,* and *RUNDC1*). **G** Venn diagram identifying 20 high-confidence hub genes that are transcriptionally upregulated, associated with open chromatin, and targeted by enhancers. **H** Detailed multi-omics view of the *FOSL2* and *KLF6* loci, demonstrating how distal sEnhs with TF footprints govern the expression of these key regulatory hubs

To elucidate the interplay between chromatin accessibility, H3K27ac, and gene expression, we integrated DARs, DPs, and RNA-seq datasets. A total of 650 genes were jointly targeted by DARs and DPs—primarily located in intronic (41.9%), distal intergenic (31.37%), and promoter regions (15.18%), consistent with enhancer localization (Fig. 8A, Table S29). About 65.59% of DEGs contained ATAC-seq peaks in their promoter or gene body, while 13.65% overlapped with H3K27ac DPs (Fig. 8B). Among DEGs, 172 were co-regulated by both mechanisms (Fig. 8C, Table S30), indicating strong epigenetic coordination. These genes were enriched in processes such as epithelial cell differentiation, binding, embryonic development, chromatin looping, and immune regulation, as well as in pathways like NF-κB, JAK-STAT, FoxO, AMPK, and HIF-1 signaling (Fig. 8D and E, Tables S31–S32). Representative hub genes—*TRAF1*, *FOSL2*, *IL6R*, *HNF1A*, and *HNF1B—*emerged as key regulators of uterine receptivity and blastocyst implantation (Fig. 8F, Table S30). Among these, sEnh-associated genes (*FOSL2*, *CEBPD*, *IL6R*, *MCL1*, *KLF6*, *IFI6*, *SUSD6*, and *MAFF*) were significantly upregulated in ERE tissues, promoting hormone responsiveness, immune tolerance, and epithelial–stromal interaction (Fig. 8G, Table S33). Conversely, genes involved in ECM remodeling and RNA stability (*CXXC5*, *DST*, *CELF2*, *LOXL1*, and *NAV1*) were downregulated, reflecting structural stabilization during receptivity (Fig. S5, Table S33). Genome browser visualization confirmed active tEnhs and sEnhs at *FOSL2*, *KLF6*, *DHX58*, *MCL1*, *IFI6*, and *SDC4* loci specifically in receptive uteri (Fig. 8H, Fig. S6). Motif analysis identified enrichment of HNF1A, JUNB, and FOSL2 binding motifs within these enhancers, suggesting direct transcriptional activation of key receptivity genes. Together, these findings reveal that coordinated remodeling of chromatin accessibility and H3K27ac establishes a super-enhancer–driven regulatory network. This epigenomic architecture orchestrates transcriptional programs governing immune tolerance, epithelial remodeling, and metabolic adaptation—molecular hallmarks of a functionally receptive endometrium prepared for blastocyst implantation.

## Discussion

As a major agricultural species, goat provide valuable sources of meat, milk, and fiber for human consumption [44]. The Dazu black goats, native to Southwest China, is highly prolific and exhibit superior reproductive traits, including high ovulation rates, low embryonic mortality, and a well-developed uterine environment [45]. In ruminants, peri-implantation development is characterized by blastocyst elongation and extensive trophoblast proliferation between 15 and 18 d post-mating [46]. In goats, this process begins around 16 d and culminates in superficial, non-invasive implantation by 18 d [47]. Consistent with these observations, our data confirm that d 17 represents the peri-implantation period in Dazu black goats. During this critical window, IFN-τ serves as the key embryonic signal for maternal recognition of pregnancy [48], regulating endometrial remodeling through interferon-responsive genes [49]. Because commercial ELISA kits for caprine IFN-τ are unavailable, we evaluated its biological activity indirectly through the expression of interferon-stimulated genes *ISG15* and *MX1*, both of which were markedly upregulated in pregnant endometria. These findings confirm active embryonic IFN-τ signaling, consistent with previous reports in ruminants [36, 37]. The establishment of endometrial receptivity occurs under P_4_ dominance, which downregulates both estrogen receptor alpha (*ESR1*) and progesterone receptor (*PGR*) in the luminal epithelium [50, 51]. This hormonal shift promotes stromal differentiation and enables uterine responsiveness to embryo-derived IFN-τ, which, together with P_4_, coordinates epithelial remodeling and immune modulation necessary for implantation [52, 53]. Several downstream genes—*MUC1*, *LTF*, *HAND2*, and *LIF—*are well-established receptivity markers. Leukemia inhibitory factor (*LIF*) has been positively correlated with implantation success, and melatonin enhances early pregnancy receptivity through the MT2/PI3K/LIF pathway [54]. A hallmark of the receptive endometrium is the plasma membrane transformation (PTM), characterized by a decreased E-cadherin/N-cadherin ratio. This molecular remodeling weakens epithelial tight junctions, facilitating blastocyst adhesion [55]. In our study, the ERE group exhibited both morphological and molecular features of receptivity, including filamentous conceptuses, mature pinopodes, higher P_4_ and IFN-τ levels, a reduced E-cadherin/N-cadherin ratio, and elevated expression of *SPP1*, *VEGF*, *LIF*, and *HAND2*, confirming the successful establishment of ERE [56].

Endometrial receptivity requires extensive transcriptional reprogramming to coordinate conceptus recognition, immune tolerance, and stromal-epithelial crosstalk. Our transcriptomic data revealed numerous DEGs involved in these processes. Among the upregulated DEGs, *SPP1*, a conserved adhesion molecule, facilitates embryo-epithelium attachment [57]. *FOXO1*, a downstream effector of progesterone signaling, promotes decidualization and oxidative stress resistance [58], while *STAT1* mediates cytokine-driven immune modulation and uterine remodeling [59]. Additionally, *CEBPD* and *KLF6* were elevated, both contributing to epithelial remodeling and mesenchymal transition [60, 61]. Interferon-stimulated genes such as *IFIT3*, *IFI6*, *ISG15*, *MX1*, and *RSAD2* were strongly induced, confirming active IFN-τ–induced immune signaling [62, 63]. Expression of *KLF4* has been linked to cell adhesion and vascular development in the uterus [64], and *KLF6* may play a similar role in vascular maturation [65]. Importantly, *NR1D1* has been shown to inhibit cell proliferation via autophagy [66], consistent with the growth arrest required for the receptive phase. Conversely, *ESR1*, *PGR*, *SLC9A5*, *SLC6A1*, *TGFB1/2*, *DAB2*, and *THBS2* were downregulated, reflecting suppression of estrogen signaling and epithelial proliferation. Suppression of *ESR1* is essential for establishing the progesterone-dominant environment of the receptive endometrium, as its downregulation relieves inhibition of *FOXO1* and activates downstream receptivity genes [67]. Similarly, *PGR* downregulation reflects the progesterone-dominant state essential for receptivity [68]. This pattern supports the “functional withdrawal” model of progesterone signaling proposed by Da Silva et al. [69], which permits *FOXO1*-mediated gene activation.

Chromatin accessibility is fundamental for transcriptional activation, enabling TFs to access regulatory DNA and establish a permissive chromatin landscape [70]. Our ATAC-seq data revealed that accessible regions were enriched near TSSs, consistent with active transcription [71]. More than 750 DEGs, including *SPP1*, *FOXO1*, *BMP6*, and *STAT1*, exhibited concurrent promoter opening and transcriptional upregulation, and were linked to immune modulation, stromal-epithelial interactions, and implantation. Similar chromatin dynamics have been reported in bovine and porcine models, where open chromatin at implantation loci predicts uterine competency [71, 72]. Integrated analysis identified several TFs with enriched chromatin footprints and elevated expression, including HNF1A, HNF1B, AP-1 complex members (FOS, FOSL1, FOSL2, and JUN), SOX13, PAX1, and BATF3, suggesting their central roles in receptivity regulation. Consistent with our observations, Maurya et al. [73] demonstrated that abnormal patterns of key receptivity markers in the endometrium arise during uterine epithelial reprogramming, underscoring the role of HNF1A in orchestrating cellular differentiation and tissue remodeling necessary for implantation. As a developmental regulator, HNF1A supports cell survival and differentiation, and its related family members contribute to the maturation of embryonic and extraembryonic lineage [74]. Vrljicak et al. [75] identified HNF1B as one of the most dynamically expressed TFs in the human endometrium, showing particularly high levels in the glandular epithelium during the implantation window. Krala et al. [76] further confirmed that HNF1B maintains the structural integrity of the uterine epithelium. In addition, elevated activity of AP-1 complex components (FOS, FOSL1, FOSL2, and JUN) suggests the presence of a hormone-responsive regulatory axis. Progesterone has been shown to upregulate c-Fos/c-Jun, thereby enhancing protein O-fucosyltransferase 1 (poFUT1) expression, which promotes embryonic adhesion [77]. FOSL1 regulates several matrix-remodeling genes (*MMP1*, *MMP2*, *MMP9*) that are essential for decidualization and implantation [78]. Notably, reduced FOSL1 expression in infertile patients has been linked to dysregulated IL-17 signaling, underscoring its critical role in immune-structural coordination within the endometrium [79]. Together, these findings suggest that HNF1A/B and AP-1 family members cooperatively contribute to the transcriptional reprogramming necessary for endometrial receptivity, although further functional validation is warranted. Interestingly, the reduced chromatin footprints of NFYA and MLX observed in receptive tissues may indicate a release of transcriptional repression at specific genomic loci, consistent with enhancer derepressing mechanisms reported during bovine embryo development [80].

Reprogramming factors can convert transcriptionally silent chromatin into an active state by recruiting chromatin remodelers and transcriptional complexes [81, 82]. Among these mechanisms, sEnhs—clusters of enhancers densely occupied by TFs—function as central hubs of gene activation and are critical for cell- and tissue-specific transcriptional regulation [83]. In this study, we identified sEnhs based on H3K27ac enrichment using the ROSE algorithm, acknowledging that their formation and activity can vary across biological contexts, as described by Blobel et al. [84]. A total of 714 newly activated sEnhs were detected in the receptive endometrium, predominantly associated with genes involved in immune regulation, hormone signaling (e.g., *IL6R*, *FOSL2*), and placental development. These findings suggest that enhancer-dependent activation underlies transcriptional programs essential for establishing endometrial receptivity. Recent studies have demonstrated that sEnhs broadly control genes related to cell proliferation, differentiation, and tissue identity [85, 86]. Consistent with these observations, our functional enrichment analysis revealed that sEnh-associated DEGs were significantly enriched in the JAK-STAT, PI3K-AKT, NF-κB, HIF-1, and extracellular matrix remodeling pathways—key biological processes governing immune regulation, decidualization, conceptus recognition, and implantation. The similarity in enhancer architectures and pathway enrichment between goats, pigs, and sheep [72, 87] indicates a conserved role of enhancer remodeling in regulating endometrial receptivity across ruminant species.

**Fig. 9:**
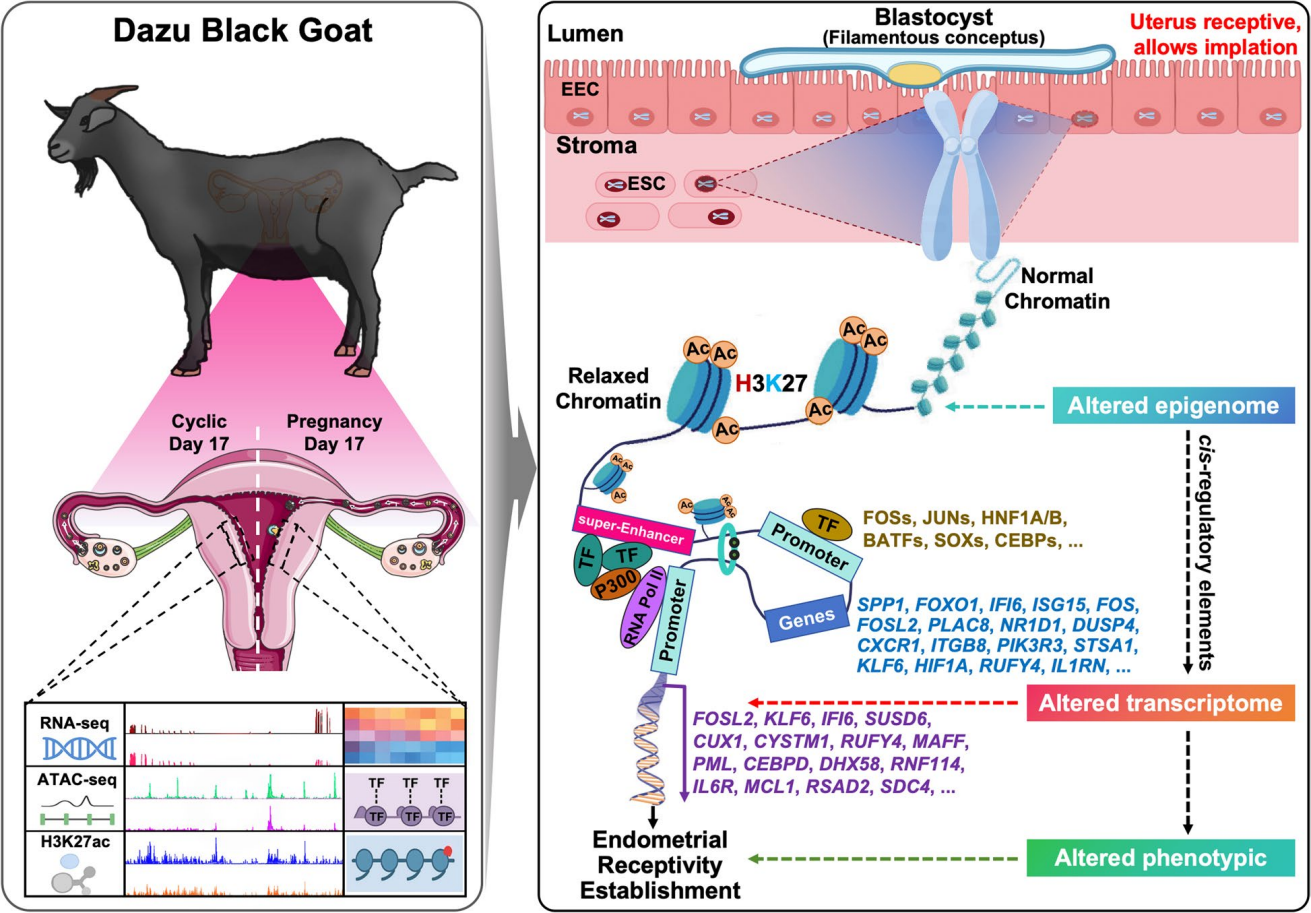
Proposed model of epigenetic regulation underlying endometrial receptivity. Schematic illustration summarizing how coordinated TFs activity, chromatin accessibility, and specific histone acetylation (H3K27ac) converge to drive expression of key receptivity genes. This model underscores an integrated epigenetic–transcriptional mechanism that establishes a uterine environment conducive to blastocyst implantation. Some of the graphic elements were created by BioRender and FigDraw. Abbreviations: EEC, endometrial epithelium cell; ESC, endometrial stromal cell; Ac, acetylation; TF, transcription factor

To further explore the coordination between chromatin accessibility and enhancer activity, we performed an integrative multi-omics analysis. This revealed a core set of 172 genes under dual epigenetic regulation, in which both open chromatin and H3K27ac modification jointly promoted transcriptional activation. Notably, several previously unreported genes—such as *CEBPD*, *MAFF*, and *NAV1—*emerged as potential regulators of endometrial remodeling and implantation. A high degree of co-occupancy between ATAC-seq peaks and H3K27ac signals was observed in enhancer-rich genomic regions, supporting a cooperative regulatory model in which accessible chromatin stabilized by H3K27ac sustains transcriptional activity. This is consistent with recent findings in sheep showing that tissue-specific H3K27ac-marked enhancers play a pivotal role in endometrial receptivity [87]. Functionally, these co-regulated genes were enriched in the PI3K-AKT, JAK-STAT, and NF-κB pathways, underscoring their essential roles in immune modulation, cell adhesion, and tissue remodeling [72]. Furthermore, several TFs enriched during the receptive phase—such as *CEBPB*, *FOSL2*, *KLF6*, *CUX1*, and *MAFF*—were themselves regulated by newly gained sEnhs, suggesting the existence of autoregulatory feedback loops between TFs and their enhancer elements. Similar chromatin-TF feedback mechanisms have been reported in embryonic stem cells and lineage differentiation systems [32, 88]. Interestingly, sEnhs located upstream of *FOSL2* and *KLF6* likely drive their elevated expression during receptivity through Mediator-mediated looping. The functional importance of these regulatory hubs is further supported by the significant enrichment of HNF1A, JUNB, JUND, and FOSL2 motifs within sEnh regions. While our data provide novel insights into how key sEnh–TF networks orchestrate endometrium–specific transcriptional activation during endometrial receptivity in goats (Fig. 9), the causal relationship between enhancer-TF interactions and gene activation requires further validation using CRISPR interference (CRISPRi) or enhancer deletion approaches.

## Conclusions

In summary, this study provides the first integrated epigenomic framework of endometrial receptivity in goats. Through synergistic multi-omics analysis, we demonstrate that chromatin accessibility and H3K27ac cooperatively activate transcriptional programs controlling cell adhesion, immune tolerance, and embryo attachment. Moreover, we identified several core transcription factors (e.g., FOSL2, KLF6, HNF1A, CEBPB) and their associated super-enhancers as major regulators of endometrial receptivity establishment. These findings offer new insights into the molecular basis of uterine receptivity and provide valuable targets for improving reproductive efficiency and fertility management in ruminant livestock.

## Supplementary Information

Additional file 1: Fig. S1. Western blotting image of E-cadherin and N-cadherin. Fig. S2. ATAC-seq and CUT&Tag quality control and data analysis in the endometrium tissue. Fig. S3. KEGG pathway analysis of DARs and DPs. Fig. S4. Box plot of the expression levels of DP-related genes. Fig. S5. Venn diagram of downregulated genes, differential ATAC-seq peaks, and enhancer targets. Fig. S6. Detailed multi-omics view of gene locus.

Additional file 2: Table S1. Details of primer sequences used for RT-qPCR. Table S2. Overview of RNA-seq data. Table S3. Overview of ATAC-seq and CUT&Tag data. Table S4. List of DEGs. Table S5. The transcription factor prediction of DEGs. Table S6. K-means clustering analysis of DEGs. Table S7. KEGG enrichment of DEGs. Table S8. Gene annotation associated with Gain DARs. Table S9. Gene annotation associated with Loss DARs. Table S10. KEGG enrichment of promoter gain DARs. Table S11. KEGG enrichment of promoter loss DARs. Table S12. Gene annotation associated with Gain DPs. Table S13. Gene annotation associated with Loss DPs. Table S14. KEGG enrichment of gain DP target genes. Table S15. Motif enrichment results in gain and loss DARs. Table S16. ATAC-seq peaks located on the DEGs' promoter and gene body. Table S17. Integrated analysis of ATAC-seq and RNA-seq data. Table S18. KEGG enrichment of up- and down-DEGs in the gain accessible groups. Table S19. CON_ATAC-seq vs. ERE_ATAC-seq. Gain. DAR. Motif annotation. Table S20. HINT TF footprinting analysis. Table S21. Identification of enhancers. Table S22. Analysis of enhancers and sEnhs target genes across CON and ERE samples. Table S23. GO enrichment analysis of sEnh-targeted DEGs in the CON and ERE group. Table S24. KEGG enrichment of up- and down-DEGs in the DP groups. Table S25. H3K27ac peaks are located on the DEGs promoter and gene body. Table S26. Overlap of the known DEGs and the genes associated with the DPs. Table S27. KEGG analysis of overlap genes. Table S28. Overlap of the gain DP and enhancer target DEGs in the ERE and CON group. Table S29. Overlap peaks of DAR and DP. Table S30. Statistics of DEGs regulated by chromatin accessibility and H3K27ac. Table S31. GO enrichment analysis of DEGs regulated by chromatin accessibility and H3K27ac. Table S32. KEGG analysis of DEGs regulated by chromatin accessibility and H3K27ac. Table S33. Overlap DEGs of DARs, DPs, and the enhancers targeted.

Additional file 3. Supplementary methods.

## Data Availability

All data analyzed during this study are available in the article and/or supporting information; further inquiries can be directed to the corresponding author on reasonable request.

## Abbreviations

ATAC-seq: Assay for transposase-accessible chromatin using sequencing
CON: Control group
CUT&Tag: Cleavage under targets and tagmentation
DAR: Differentially accessible regions
DEG: Differentially expressed gene
DP: Differential peaks
E_2_: 17β-Estradiol
EEC: Endometrial epithelium cell
Enh: Enhancer
ERE: Endometrial receptivity
ESC: Endometrial stromal cell
FAANG: Functional Annotation of Animal Genomes
FarmGTEx: Farm Animal Genotype-Tissue Expression
FDR: False discovery rate
FIMO: Find Individual Motif Occurrences
GO: Gene Ontology
GTF: General transcription factor
IDR: Irreproducible discovery rate
IFN-τ: Interferon-tau
IVF: In-vitro fertilization
KEGG: Kyoto Encyclopedia of Genes and Genomes
MEME: Multiple Em for Motif Elicitation
P_4_: Progesterone
PMT: Plasma membrane transformation
RNA-seq: RNA-sequencing
RT-qPCR: Real-time quantitative PCR
SEM: Scanning electron microscope
sEnh: Super-enhancer
tEnh: Typical enhancer
TF: Transcription factor
TSS: Transcription start sites
UTR: Untranslated Regions
