# Supplementary material for: Multi-omics profiling of chromatin accessibility and H3K27ac reveals super-enhancer–mediated regulatory networks governing endometrial receptivity in goats
Source: J Anim Sci Biotechnol. 2026 Jan 9;17:4. doi: 10.1186/s40104-025-01318-2 (PMC12784515; doi:10.1186/s40104-025-01318-2)
Supplement: Supplementary file 1 — Additional file 1: Fig. S1. Western blotting image of E-cadherin and N-cadherin. Fig. S2. ATAC-seq and CUT&Tag quality control and data analysis in the endometrium tissue. Fig. S3. KEGG pathway analysis of DARs and DPs. Fig. S4. Box plot of the expression levels of DP-related genes. Fig. S5. Venn diagram of downregulated genes, differential ATAC-seq peaks, and enhancer targets. Fig. S6. Detailed multi-omics view of gene locus. [file 40104_2025_1318_MOESM1_ESM.docx]

**Supplementary Figures**


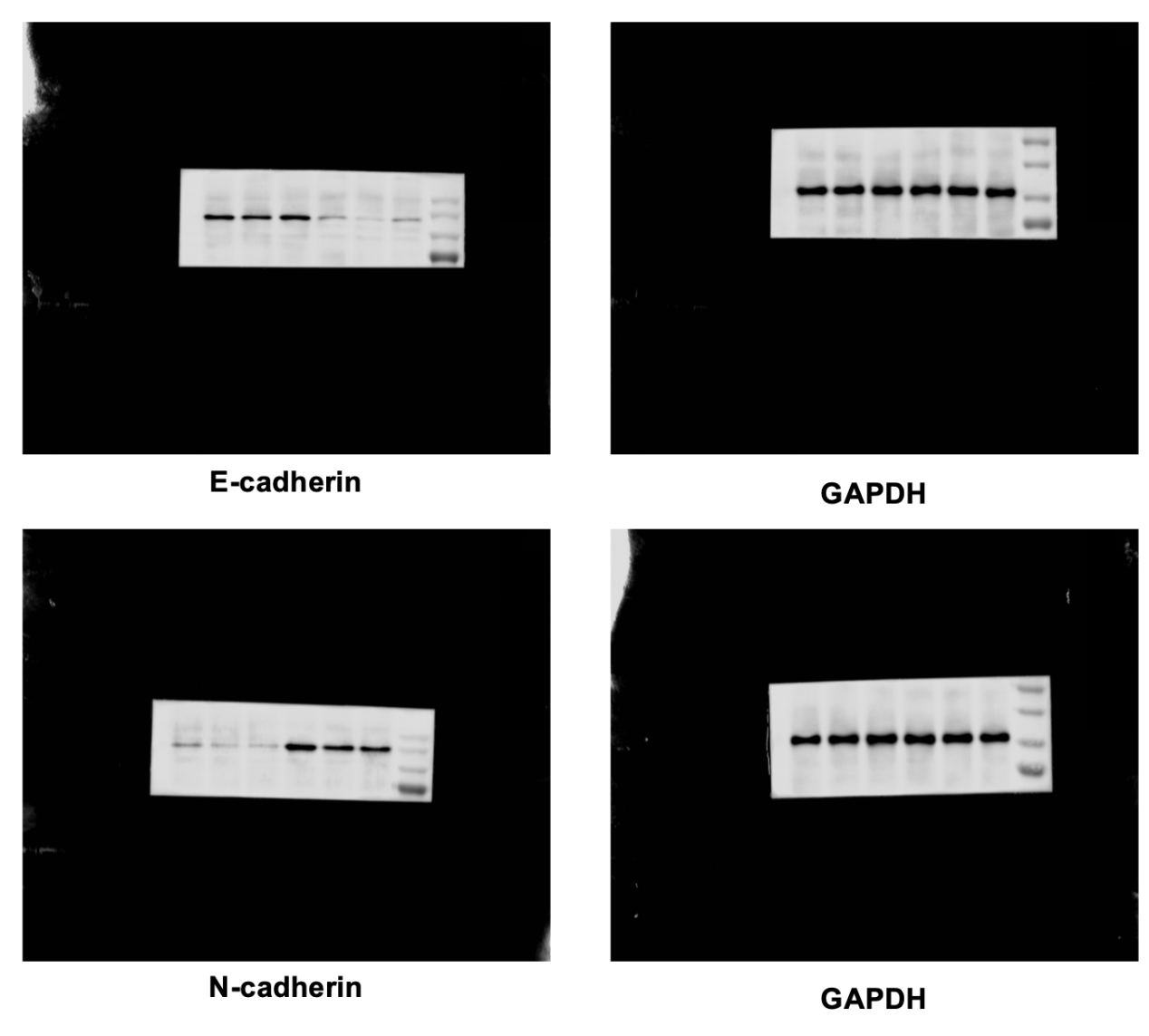


**Figure S1.** Western blotting image of E-cadherin and N-cadherin.


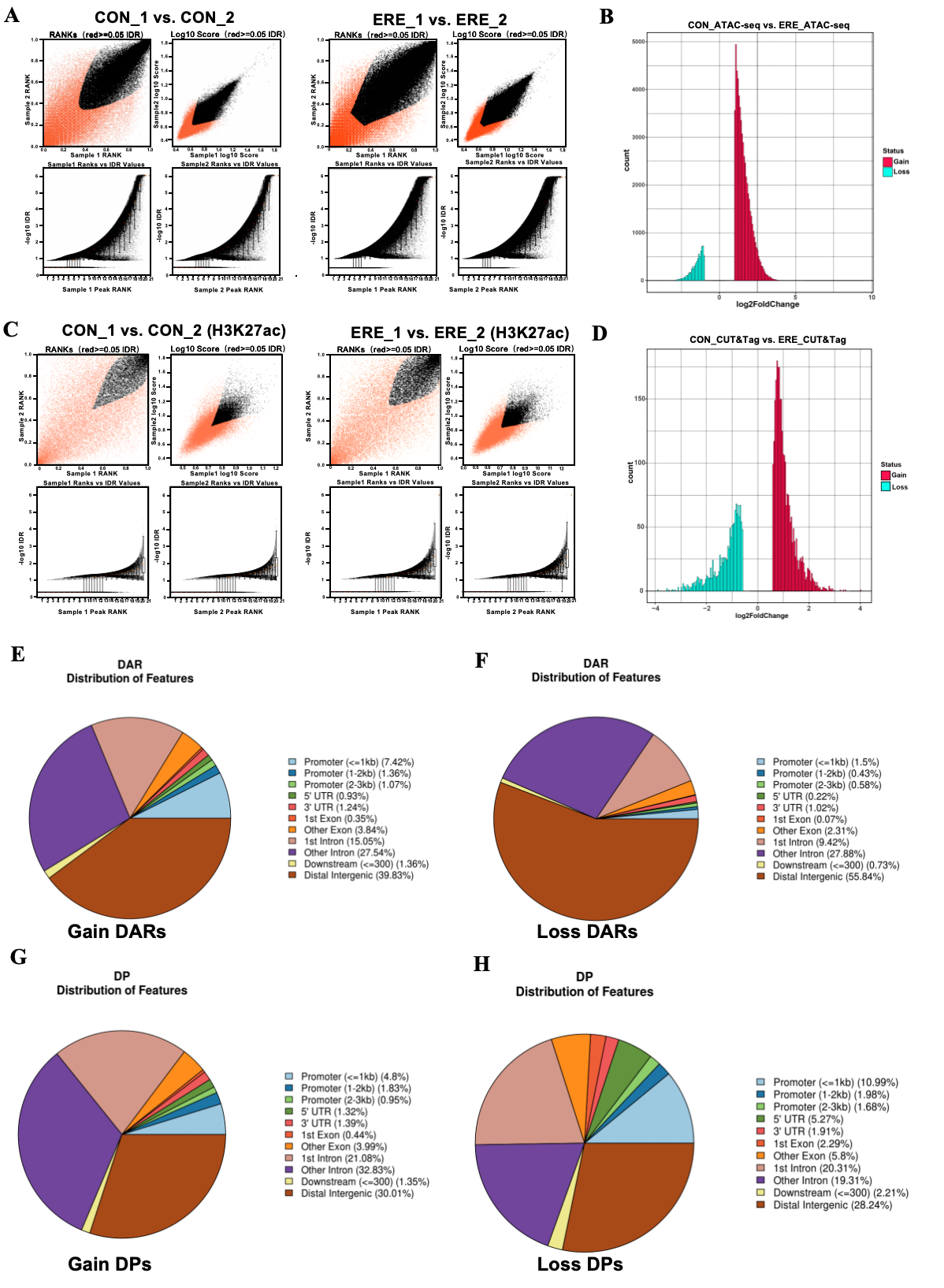


**Figure S2.** ATAC-seq and CUT&Tag quality control and data analysis in the endometrium tissue. **A** and **C** IDR analysis of reproducibility within groups. **B** and **D**Histogram of DARs (**B**) and DPs (**D**). **E** and **F** Genome-wide distribution of gain (**E**) and loss (**F**) DARs. **G** and **H** Genome-wide distribution of gain (**G**) and loss (**H**) DPs.

**
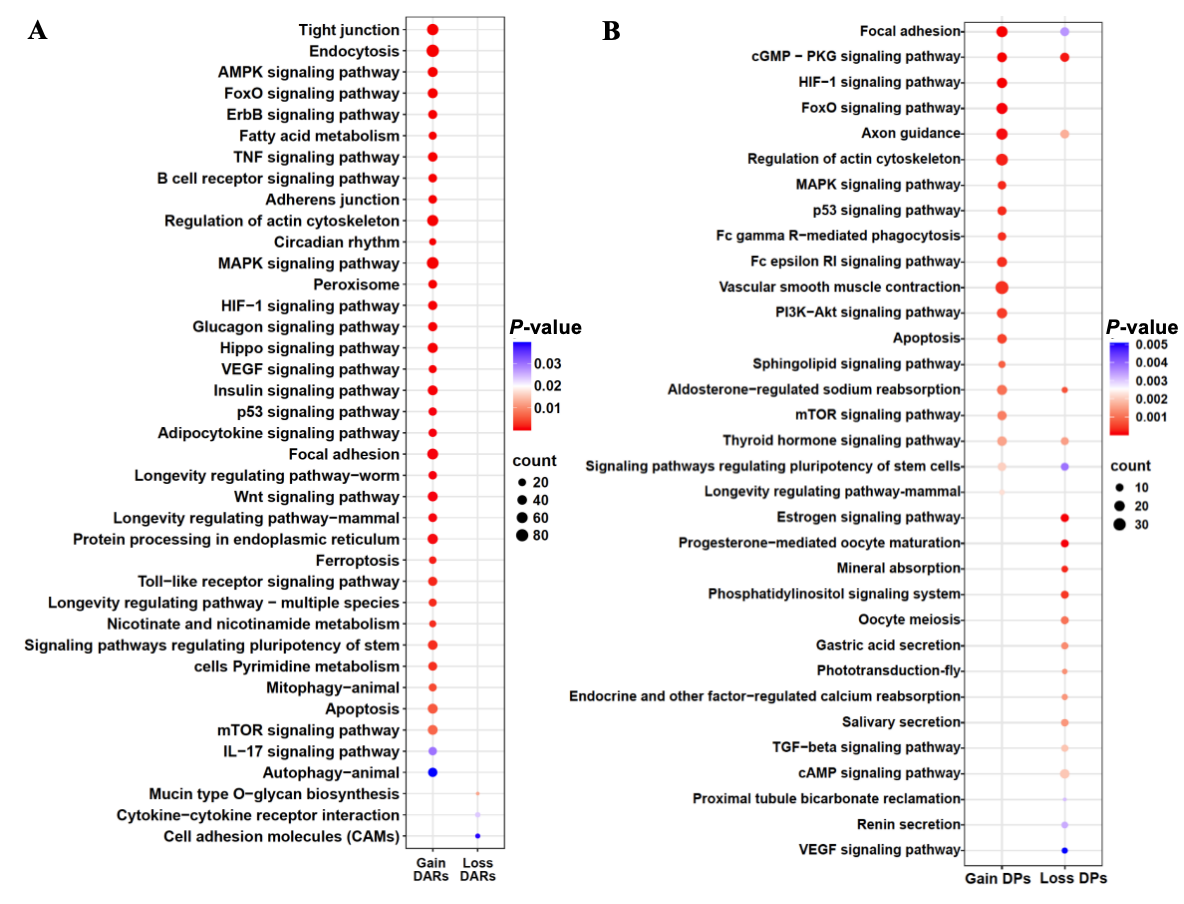
**

**Figure S3.** KEGG pathway analysis of DARs and DPs. **A** KEGG pathway analysis of gain and loss promoter DAR associated genes. **B** KEGG pathway analysis of gain and loss DP associated genes.


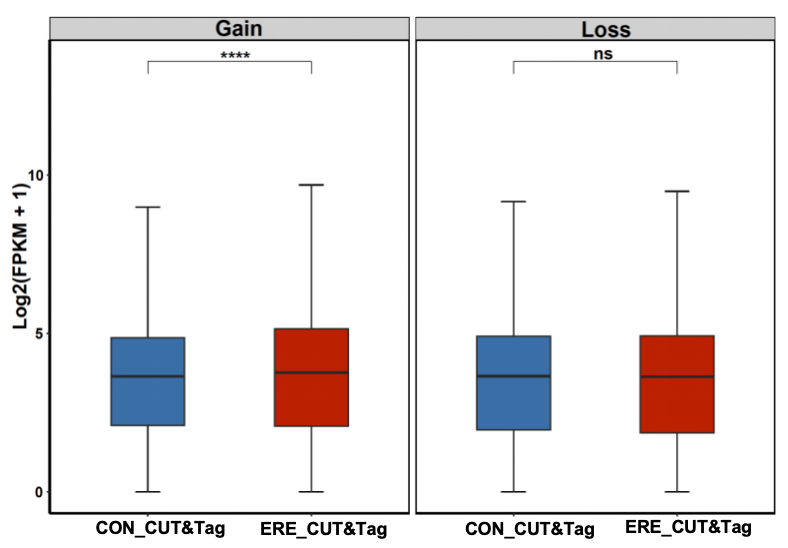


**Figure S4.** Box plot of the expression levels of DP-related genes.


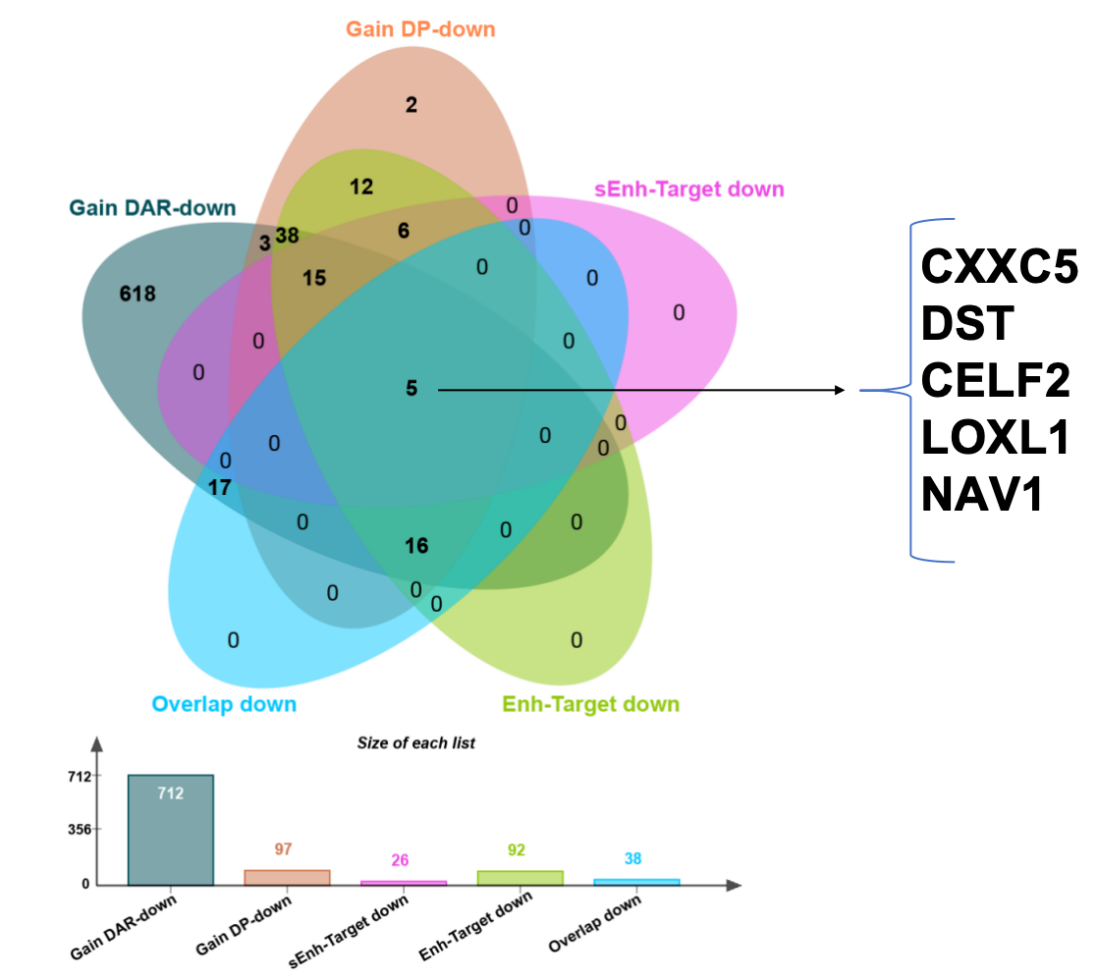


**Figure S5**. Venn diagram of downregulated genes, differential ATAC-seq peaks, and enhancer targets.


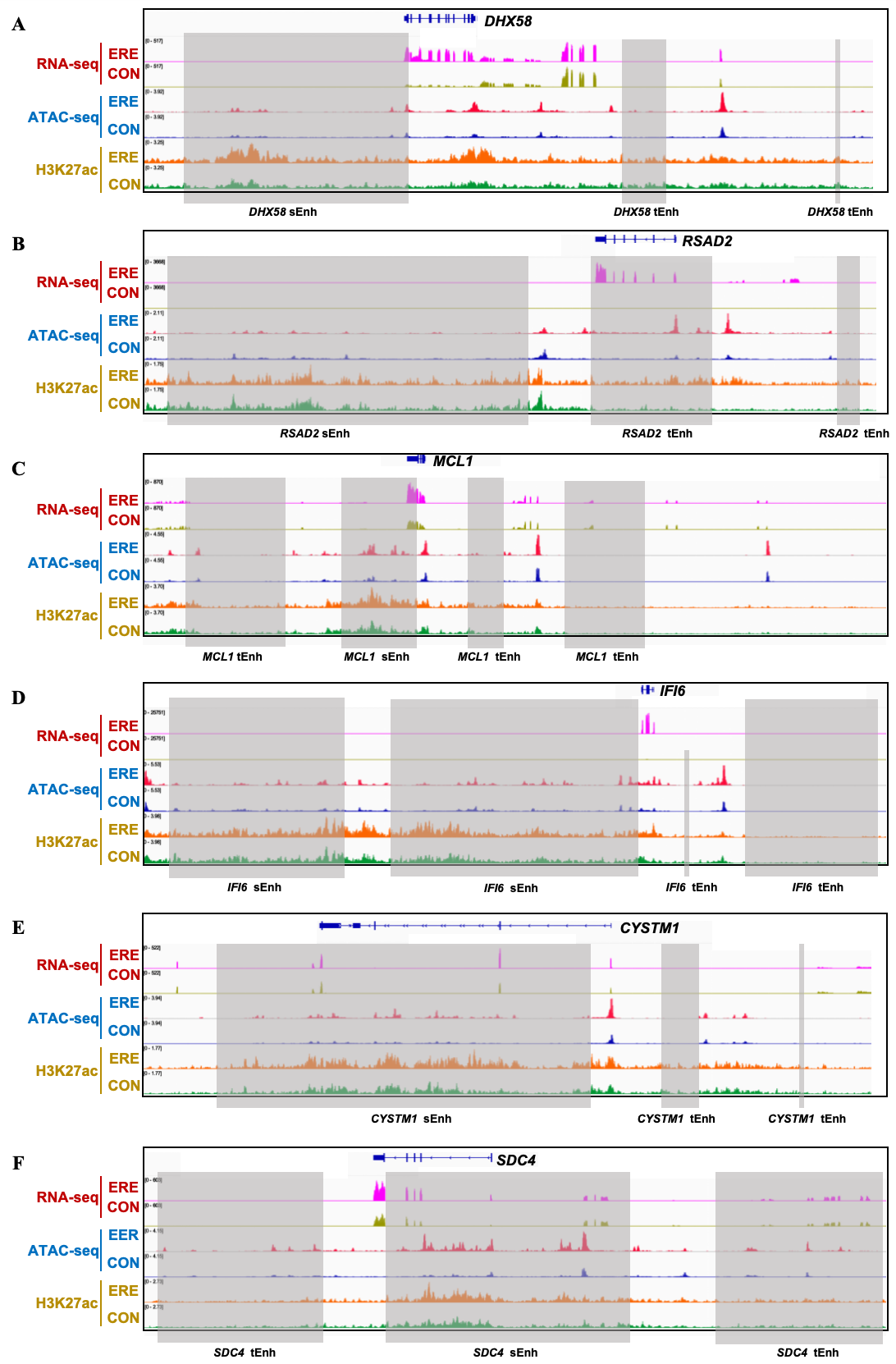


**Figure S6**. Detailed multi-omics view of genes locus. **A-F** ATAC-seq, H3K27ac, and RNA-seq profile at the *DHX58* (**A**), *RSAD2* (**B**), *MCL1* (**C**), *IFI6* (**D**), *CYSTM1* (**E**), and *SDC4* (**F**) locus. Grey boxes indicate sEnh and tEnh identified.
