# Supplementary material for: Multi-omics profiling of chromatin accessibility and H3K27ac reveals super-enhancer–mediated regulatory networks governing endometrial receptivity in goats
Source: J Anim Sci Biotechnol. 2026 Jan 9;17:4. doi: 10.1186/s40104-025-01318-2 (PMC12784515; doi:10.1186/s40104-025-01318-2)
Supplement: Supplementary file 3 — Additional file 3. Supplementary methods. [file 40104_2025_1318_MOESM3_ESM.docx]

**Supplementary Methods**

**Enzyme-linked immunosorbent assay (Elisa)**

To determine serum progesterone (P_4_) concentrations, blood samples were collected from goats on Day 17 of pregnancy or non-pregnancy via jugular venipuncture using 10 mL vacuum tubes without anticoagulant. Samples were allowed to clot at room temperature for 30 min and then centrifuged at 3000 × g for 15 min at 4°C. The resulting serum was aliquoted and stored at –80°C until further analysis. Serum levels of P_4_ were quantified using commercial enzyme-linked immunosorbent assay (ELISA) kits specific for caprine species (Progesterone ELISA Kit, Goat; Ruixin Bio, China), following the manufacturer’s protocols. Briefly, 50 µL of diluted serum samples (1:2 in assay buffer) and standards were added to each well, followed by the addition of enzyme conjugate and anti-progesterone antibody. After incubation for 1 h at 37°C, wells were washed and incubated with TMB substrate for 15 min in the dark. The reaction was terminated with stop solution, and absorbance was measured at 450 nm using a microplate reader. All data are presented as the mean ± standard error of the mean (SEM). Statistical analyses were performed using GraphPad Prism (v10.3.1; GraphPad Software, San Diego, CA, USA), and differences were considered statistically significant at *P* < 0.05.

**Cleavage under targets and tagmentation (CUT&Tag)**

For CUT&Tag, nuclei were isolated from flash-frozen tissues following the same procedure as used for ATAC-seq. The assay was performed by Frasergen (Wuhan, China). Briefly, 100,000 cells were collected and centrifuged at 600 × g for 3 min at room temperature, followed by two washes with 300 μL of Wash Buffer (20 mmol/L HEPES, pH 7.5; 150 mmol/L NaCl; 0.5 mmol/L spermidine; 1× protease inhibitor cocktail).

Next, 10 μL of activated concanavalin A–coated magnetic beads was added to the cell suspension and incubated at room temperature for 10 min. After removing the supernatant, bead-bound cells were resuspended in 50 μL of Dig-Wash Buffer (20 mmol/L HEPES, pH 7.5; 150 mmol/L NaCl; 0.5 mmol/L spermidine; 1× protease inhibitor cocktail; 0.05% digitonin) containing 2 mmol/L EDTA and a 1:50 dilution of the primary antibody (anti-H3K27ac). The suspension was gently rotated for 2 h at room temperature.

After magnetic separation, cells were incubated with 50 μL of a 1:50 dilution of goat anti-rabbit IgG secondary antibody (Proteintech, B900210) in Dig-Wash Buffer for 1 h at room temperature. For negative controls, only the secondary antibody was used. Cells were then washed twice (1 min each) with 500 μL of Dig-Wash Buffer.

A 1:200 dilution of the pG-Tn5 adapter complex (~0.04 μmol/L) was prepared in Dig-300 Buffer (0.01% digitonin; 20 mmol/L HEPES, pH 7.5; 300 mmol/L NaCl; 0.5 mmol/L spermidine; 1× protease inhibitor cocktail), and 100 μL of this solution was added to the cells with gentle vortexing. After incubation for 1 h at room temperature, cells were washed twice (1 min each) with 500 μL of Dig-300 Buffer to remove unbound pG-Tn5.

Subsequently, cells were resuspended in 300 μL of Tagmentation Buffer (10 mmol/L MgCl₂ in Dig-300 Buffer) and incubated at 37 °C for 1 h. The tagmentation reaction was terminated by adding 10 μL of 0.5 M EDTA, 3 μL of 10% SDS, and 2.5 μL of 20 mg/mL Proteinase K, followed by incubation at 55 °C for 1 h. DNA was purified by phenol–chloroform–isoamyl alcohol extraction, washed with ethanol, and resuspended in nuclease-free water.

PCR amplification was performed with an initial extension at 72 °C for 3 min, denaturation at 98 °C for 30 s, followed by 16 cycles of 98 °C for 15 s, 60 °C for 30 s, and 72 °C for 30 s, and a final extension at 72 °C for 3 min. The amplified DNA was purified using Ampure XP beads (Beckman Coulter) and sequenced on an Illumina NovaSeq platform.

**ATAC-seq and CUT&Tag data analysis**

For ATAC-seq and H3K27ac CUT&Tag data, raw reads were first processed using Trimmomatic (v0.39) to remove adapter sequences, low-quality bases, and undetected bases (N). The parameters used were: LEADING:3 TRAILING:3 SLIDINGWINDOW:4:15 MINLEN:8. Quality assessment of paired-end reads was performed with FastQC (v0.11.9). Clean reads were aligned to the reference genome using Bowtie2 (v2.3.5) with the following parameters: --dovetail --no-unal --very-sensitive-local --no-mixed --no-discordant. Low-quality alignments, PCR duplicates, and organelle-derived reads were removed using Samtools (v1.12) and Picard (v2.25.6) with default settings to obtain high-confidence valid pairs for downstream analysis.

Peak calling was performed using MACS3 (v3.0.0a6) based on a statistical model with parameters -f BAMPE -B -SPMR --keep-dup all. For experiments with biological replicates, overlapping peaks between replicates were identified to evaluate data reproducibility.

Signal enrichment around peak regions, gene bodies, and transcription start sites (TSSs) was analyzed using deepTools (v3.5.1) in R. Gene annotation of peak regions was performed using ChIPseeker. To visualize genomic enrichment and peak distributions, representative regions were randomly selected and plotted using Gviz. The WashU Epigenome Browser (http://epigenomegateway.wustl.edu/browser/) was used to visualize H3K27ac CUT&Tag and gene expression profiles. Finally, correlations among read distributions from RNA-seq, ATAC-seq, and CUT&Tag datasets across different tissues were evaluated using Pearson correlation coefficients.
